# Iron catalyzed CO_2_ hydrogenation to formate enhanced by Lewis acid co-catalysts†
†Electronic supplementary information (ESI) available: Experimental details and characterizing data, X-ray CIFs, and selected NMR spectra. CCDC 1061151–1061157. For ESI and crystallographic data in CIF or other electronic format see DOI: 10.1039/c5sc01467k


**DOI:** 10.1039/c5sc01467k

**Published:** 2015-05-28

**Authors:** Yuanyuan Zhang, Alex D. MacIntosh, Janice L. Wong, Elizabeth A. Bielinski, Paul G. Williard, Brandon Q. Mercado, Nilay Hazari, Wesley H. Bernskoetter

**Affiliations:** a The Department of Chemistry , Brown University , Providence , RI 02912 , USA . Email: wb36@brown.edu; b The Department of Chemistry , Yale University , New Haven , CT 06520 , USA . Email: nilay.hazari@yale.edu

## Abstract

A family of iron(ii) carbonyl hydride complexes supported by either a bifunctional PNP ligand containing a secondary amine, or a PNP ligand with a tertiary amine that prevents metal–ligand cooperativity, were found to promote the catalytic hydrogenation of CO_2_ to formate in the presence of Brønsted base. In both cases a remarkable enhancement in catalytic activity was observed upon the addition of Lewis acid (LA) co-catalysts. For the secondary amine supported system, turnover numbers of approximately 9000 for formate production were achieved, while for catalysts supported by the tertiary amine ligand, nearly 60 000 turnovers were observed; the highest activity reported for an earth abundant catalyst to date. The LA co-catalysts raise the turnover number by more than an order of magnitude in each case. In the secondary amine system, mechanistic investigations implicated the LA in disrupting an intramolecular hydrogen bond between the PNP ligand N–H moiety and the carbonyl oxygen of a formate ligand in the catalytic resting state. This destabilization of the iron-bound formate accelerates product extrusion, the rate-limiting step in catalysis. In systems supported by ligands with the tertiary amine, it was demonstrated that the LA enhancement originates from cation assisted substitution of formate for dihydrogen during the slow step in catalysis.

## Abstract 2

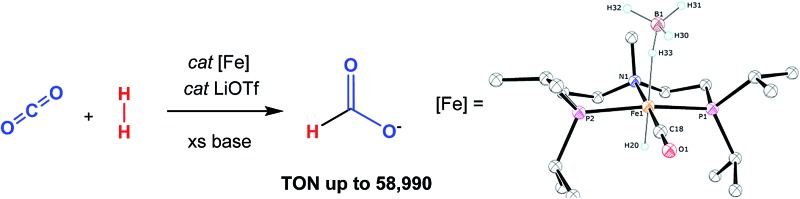
Iron/Lewis acid co-catalysts hydrogenate to CO_2_ to formate with unprecedented turnover for a first row transition metal catalyst.

## Introduction

The increasing volatility in price and the negative environmental impact associated with fossil fuel utilization for energy and commodity chemical production continues to spur basic research into the exploitation of renewable carbon resources.^1^ CO_2_ is an attractive target for transitioning the chemical industry to sustainable feedstocks, due to its incredible abundance, cheap availability and low toxicity.^2^ Formic acid is an especially interesting CO_2_ reduction product given its use in numerous agrochemicals and preservatives,^3^ as well as its potential role as a material for chemical hydrogen storage (CHS) in renewable energy applications.^4^ The utilization of formic acid as a CHS material requires reversible hydrogenation/dehydrogenation between CO_2_ and formic acid, a reaction with a small thermodynamic preference (7 kcal mol^–1^) toward CO_2_ and H_2_ in the gas phase.^5^ Consequently, most catalysts for the hydrogenation of CO_2_ to formic acid rely on exogenous base to form formate, which drives the reaction.^6^ The most effective of these catalysts employ precious metals such as ruthenium,^7^ rhodium,^8^ and iridium,^9^ and turnover numbers (TONs) of approximately 3.5 × 10^6^ and turnover frequencies (TOFs) near 150 000 h^–1^ have been achieved at elevated pressures and temperatures (49–59 atm; 120–220 °C).^10^ These findings demonstrate the remarkable potential for catalytic CO_2_ hydrogenation to formate, but also motivate the development of earth abundant catalytic systems, which are expected to enhance the sustainability and economic feasibility of this transformation.

Although homogeneous catalysts for CO_2_ hydrogenation to formate containing first-row transition metals were first described in 1976,^11^ TONs were low. As a result for many decades significantly more attention was devoted to the study of heterogeneous catalysts containing first-row transition metals.^12^ However, in recent years, the development of homogenous catalysts has been reinvigorated by the discovery of several more active cobalt and iron based systems.^13^ For example, Fujita *et al.* reported a Cp*Co (Cp* = η^5^-C_5_Me_5_) complex supported by a dihydroxy-bipyridine ligand that is capable of 59 turnovers to formate in aqueous bicarbonate,^14^ while Linehan and coworkers described an even more impressive TON of 9400 using (Me_2_PCH_2_CH_2_PMe_2_)_2_CoH, although the use of a costly and strong base (Verkade's base) is required for high conversion.^15^ During the same time period, Beller and coworkers described both cobalt and iron catalysts supported by tetraphosphine ligands, with the [P(*o*-C_6_H_4_PPh_2_)_3_Fe]^2+^ congener affording the highest TON for an iron catalyst to date.^16^ This *in situ* generated system afforded 1897 turnovers to formate in methanol/water with excess NEt_3_ under 60 atm of CO_2_/H_2_ at 100 °C.16*b* Another noteworthy iron system was reported by Milstein and coworkers who showed that a complex supported by pincer ligand with a pyridine backbone could give 708 turnovers under remarkably mild pressures (8–10 atm) in 2 M aqueous NaOH.^17^ Collectively these discoveries establish that earth abundant metals are capable of promoting CO_2_ hydrogenation, but their activities lag far beyond those of precious metal catalysts.

**Fig. 1:**
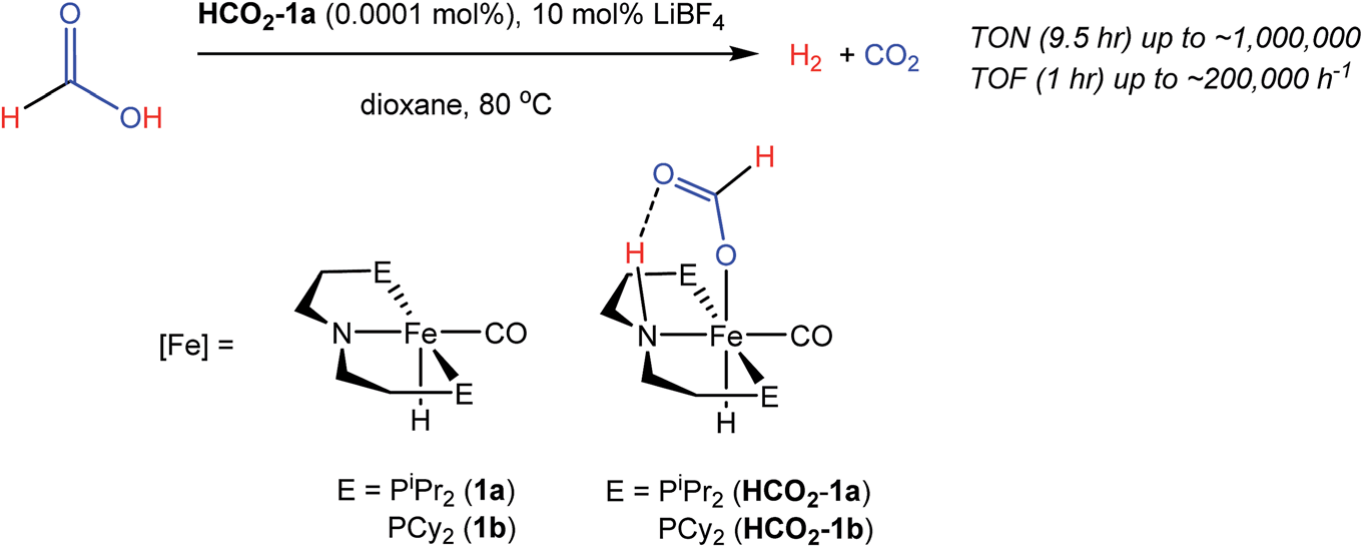
Iron–LA co-catalyzed dehydrogenation of formic acid.

Our laboratories recently identified iron catalysts for formic acid dehydrogenation (FADH), the reverse of CO_2_ hydrogenation, which surpass even precious metal catalysts in activity.^18^ The iron(ii) formate carbonyl hydride species, (^R^PNP)Fe(H)CO(HCO_2_) (^R^PNP = HN{CH_2_CH_2_(PR_2_)}_2_; R = ^i^Pr (**HCO_2_-1a**), R = Cy (**HCO_2_-1b**)), bearing a bifunctional amine ligand give 1 × 10^6^ turnovers for FADH with a TOF near 200 000 h^–1^ (Fig. 1). Slightly diminished performance was also observed using the five-coordinate iron(ii) species, ((^R^PNP)Fe(H)CO; R = ^i^Pr (**1a**), R = Cy (**1b**)), which readily form **HCO_2_-1a** and **HCO_2_-1b** upon exposure to formic acid. The impressive activity is dependent on the presence of a Lewis acid (LA) co-catalyst, such as LiBF_4_, which preliminary mechanistic studies indicate aids in the decarboxylation of an iron-formate intermediate. Herein we report the development of a collection of iron complexes based on this PNP ligand motif, which catalyze the hydrogenation of CO_2_ to formate with TONs approaching 60 000, far greater than any previously described earth abundant metal catalyst. These high TONs are only achieved in the presence of a LA co-catalyst and we describe detailed mechanistic studies which elucidate the crucial role of the LA.

## Results and discussion

### CO_2_ hydrogenation activity of (^R^PNP)Fe(H)CO

The mildly endergonic profile of CO_2_ hydrogenation to formic acid has led researchers to employ a wide variety of exogenous bases to drive this reaction. Given the limited stability of **1a** and **1b** in aqueous environments,^19^ our initial catalytic experiments focused on identifying suitable bases with moderate to good solubility in organic solvents. A brief screen of bases using **1b** in THF and a combined 69 atm of CO_2_/H_2_ (1 : 1) at 80 °C indicated that Cs_2_CO_3_ and 1,8-diazabicycloundec-7-ene (DBU) were among the most effective bases in promoting formate production (Table S1†). For subsequent investigations, DBU was selected as the base of choice owing to its higher solubility in THF and moderately better TON compared to Cs_2_CO_3_. The TON of 78 observed using DBU indicates that **1b** is, at best, a modest catalyst for CO_2_ reduction by itself. However, addition of LiBF_4_ dramatically improved the conversion.^20^ Conducting catalytic reactions using **1a** and **1b** in the presence of a LA (2 : 1 ratio of DBU : LiBF_4_) afforded a *ca.* 3–4 fold increase in TON, with the –PCy_2_ substituted **1b** showing a slightly higher conversion (Table 1). A screen of LiBF_4_ loadings between DBU : LiBF_4_ ratios of 150 : 1 to 2 : 1 showed an onset of saturation behavior below 6 : 1 (Table S2†), thus a DBU : LA ratio of 7.5 : 1 was employed as the benchmark co-catalyst loading for most catalytic experiments reported here. This loading balances the higher conversions at increased LA loadings with possible complications arising from solubility limitations.

**Table 1:** Lewis acid enhancement of CO_2_ hydrogenation catalyzed by **1a** and **1b**^*a*^

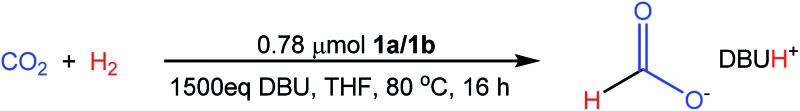				
Entry	Catalyst	DBU/LiBF_4_	TON^*b*^	Yield^*c*^ (%)
1	**1a**	No LiBF_4_	240	16
2	**1b**	No LiBF_4_	430	28
3	**1a**	2/1	1010	67
4	**1b**	2/1	1220	82

^*a*^Reaction conditions: 69 atm of CO_2_ : H_2_ (1 : 1), 0.78 μmol of **1a** or **1b** in 5 mL THF (*ca.* 0.015 M), 180 mg DBU at 80 °C.

^*b*^Formate production quantified by ^1^H NMR spectroscopy; reported values are the average of three trials.

^*c*^Reported yields are based on DBU : formate of 1 : 1.

In our previous work on LA enhanced FADH we screened a large range of Lewis acidic salts and identified LiBF_4_ as the optimum co-catalyst.^18^ A more limited examination of LAs was conducted for the CO_2_-to-formate reaction with an emphasis on using readily available alkali metal salts (Table 2). Entries 2–4 show the superior performance of the trifluoromethanesulfonate anion (OTf^–^) compared to BF_4_^–^ or Cl^–^. In the case of Cl^–^, an inhibition of the reaction compared to no added LA was observed, likely due to coordination of Cl^–^ to the iron catalyst. Additional comparison of the three lightest alkali metal OTf salts (entries 4–6) indicated good TON for all species, with a slightly higher conversion for the Li cation.

**Table 2:** Lewis acid screening for CO_2_ hydrogenation catalyzed by **1b**^*a*^

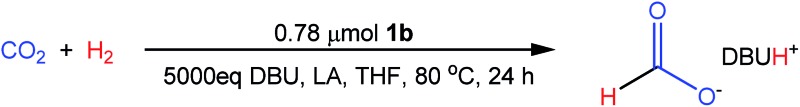				
Entry	LA	DBU/LA	TON^*b*^	Yield^*e*^ (%)
1	No LA	No LA	880	17
2	LiCl^*d*^	7.5/1	190	4
3	LiBF_4_	7.5/1	2250	45
4	LiOTf	7.5/1	3070^*c*^	61
5	NaOTf	7.5/1	2520	50
6	KOTf	7.5/1	2680	54

^*a*^Reaction conditions: 69 atm of CO_2_ : H_2_ (1 : 1), 0.78 μmol of **1b** in 5 mL THF (*ca.* 0.015 M), 600 mg DBU (3.94 mmol) at 80 °C.

^*b*^Formate production quantified by ^1^H NMR spectroscopy.

^*c*^Reported value is the average of two trials.

^*d*^LiCl was not fully soluble at ambient temperature under these conditions.

^*e*^Reported yields are based on DBU : formate of 1 : 1.

The incongruous reaction conditions employed across most iron and cobalt catalyzed CO_2_ hydrogenation reactions make a definitive comparison of catalyst activity challenging. The *ca.* 3000 TON observed for the LiOTf/**1b** catalyzed CO_2_-to-formate reaction (Table 2; entry 4) is higher than any other iron mediated system to date and it functions under comparable reaction conditions to the [P(*o*-C_6_H_4_PPh_2_)_3_Fe]^2+^ catalyst described by Beller.16*b* The LA/Fe co-catalyzed reaction is also far more active than cobalt catalysts when DBU is employed as the common base, although higher TONs are achieved with cobalt under different reaction conditions.^15^

### Synthesis and characterization of [^R^PN^Me^PFe] complexes

**Fig. 2:**
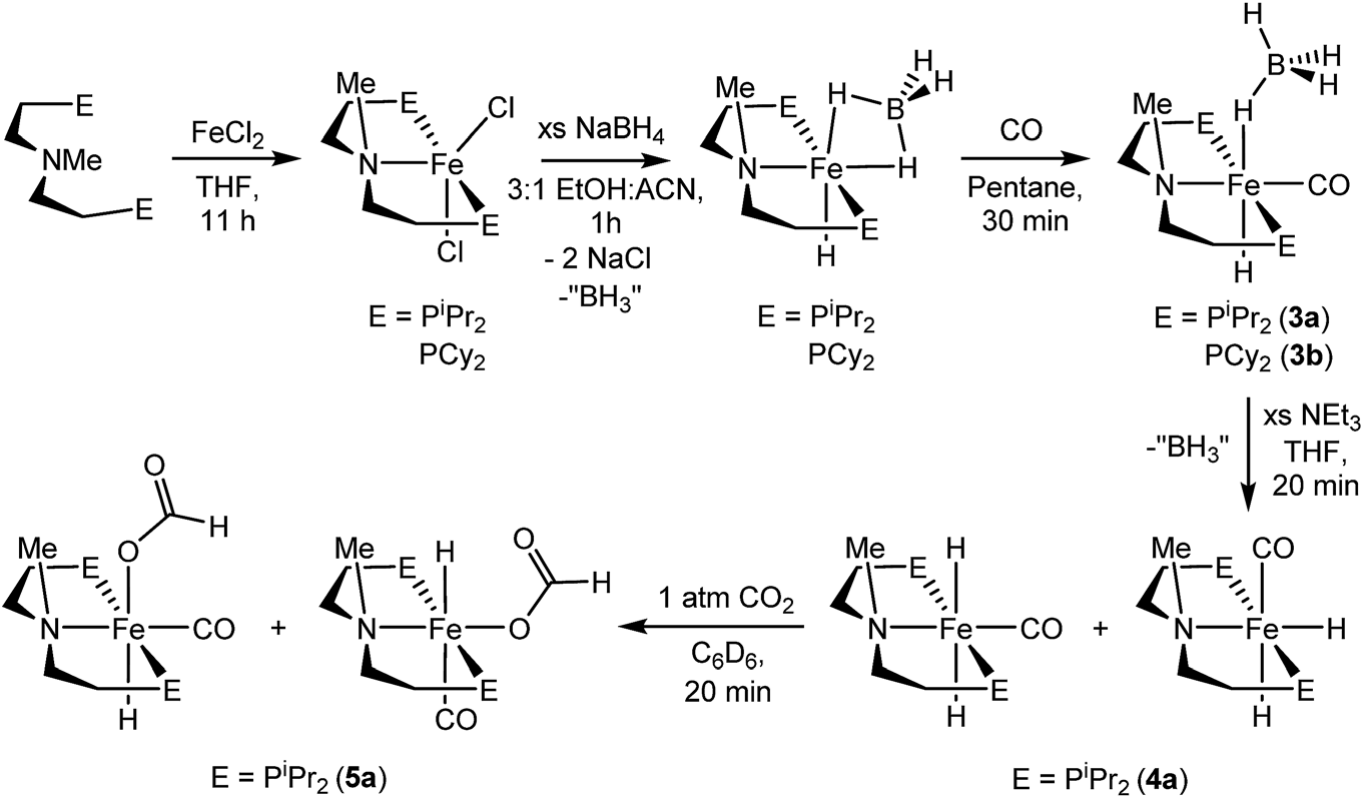
Synthesis of [^R^PN^Me^PFe] complexes.

A potentially important feature of **1** is that it can undergo reactions in which there is metal–ligand cooperation^21^ due to the ability of the PNP ligand to be in either a protonated or deprotonated form.^18^,^19^,^22^ Beller and coworkers have recently reported the synthesis of MeN{CH_2_CH_2_(P^i^Pr_2_)}_2_ and its coordination to iron as part of control experiments relating to catalytic nitrile and ester hydrogenation.^23^ To further explore the role of the bifunctional ligand we were interested in comparing the activity for CO_2_ hydrogenation of iron complexes supported by both ^R^PN^H^P and ^R^PN^Me^P ligands. In our hands both the isopropyl and cyclohexyl versions of ^R^PN^Me^P were coordinated to FeCl_2_ by stirring in THF solution to give excellent yields of (^R^PN^Me^P)FeCl_2_ (Fig. 2). Each species displays a set of broad peaks between *ca.* –5 and 75 ppm in the ^1^H NMR spectrum, indicative of a paramagnetic substance. The molecular structure of (^iPr^PN^Me^P)FeCl_2_ was confirmed by X-ray diffraction (Fig. S16†) and exhibited a distorted square pyramidal geometry (*τ* = 0.37).^24^ Treatment of these iron(ii) dichloride species with sodium borohydride in MeCN/EtOH afforded the six-coordinate (^R^PN^Me^P)Fe(H)BH_4_ species (Fig. 2). The structure of the P^i^Pr_2_ congener was characterized by X-ray diffraction as depicted in Fig. S17.† The data were of sufficient quality that all hydrogen atoms, including those bound to iron and boron, were located in the difference map and clearly indicate a κ^2^-coordination of the BH_4_ ligand.

The (^R^PN^Me^P)Fe(H)BH_4_ species each react readily with 1 atm of CO to yield (^R^PN^Me^P)Fe(H)CO(BH_4_) (R = ^i^Pr (**3a**), R = Cy (**3b**)) as yellow compounds (Fig. 2). The highest purity materials were obtained from syntheses conducted in pentane over short reaction times (30–45 minutes). ^1^H NMR spectra of **3a** and **3b** in benzene-*d*_6_ each display an iron-hydride resonance near –20 ppm and a very broad borohydride signal around –2.5 ppm. The broad resonance of the BH_4_ fragment is typical of κ^1^-coordinated species and suggests a rapid interchange of the bound B–H bond on the NMR timescale.^25^ The P^i^Pr_2_ congener, **3a**, again provided a solid state structure from X-ray diffraction experiments (Fig. S18†), which confirms the κ^1^-coordination of BH_4_ and the binding of CO ligand *trans* to the tertiary amine.

The thermal stability of **3a** in benzene or THF was limited. Even upon standing under an N_2_ atmosphere for 1 hour, new Fe–H resonances began to appear in the ^1^H NMR spectrum. These resonances, along with signals in the ^31^P NMR spectrum, are consistent with those previously described by Beller and coworkers for the *cis* and *trans* dihydride isomers of (^iPr^PN^Me^P)Fe(H)_2_CO (**4a**).^23^,^26^ The conversion of **3a** to **4a** appears to be influenced by solvent and exposure to vacuum, with use of THF and lower pressures enhancing formation of the iron(ii) dihydride species. Pure samples of **4a** were obtained by treatment of **3a** with a large excess of NEt_3_ and crystallization from pentane at low temperature. Crystal samples of **4a** obtained at –30 °C consistently afforded a molecular structure of the *cis* dihydride isomer (Fig. S19†), including characterization of two polymorphs of the material.^27^ However, solutions prepared from the crystalline material consistently show a 3 : 1 ratio favoring the *trans* dihydride isomer. EXSY NMR experiments (mixing time 800 ms at 22 °C) do not display correlations indicative of rapid isomer interconversion on the NMR timescale, but the consistent ratio from multiple samples suggests isomerization likely occurs over longer time periods.

The addition of 1 atm of CO_2_ to **4a** generated the iron formate complex **5a** as a 5 : 1 mixture of two isomers (Fig. 2). The major isomer of **5a** exhibits an Fe–H resonance at –23.89 ppm (^3^*J*_P–H_ = 52 Hz) and a formate C–H peak at 9.22 ppm in the ^1^H NMR spectrum along with a signal at 84.81 ppm in the ^31^P {^1^H} NMR spectrum. The minor isomer displays similar resonances, which are illustrated in the ESI.† A structural assignment of the isomers of **5a** was based on a combination of 2D NOESY, ^13^CO isotopic labeling and X-ray diffraction experiments. Cooling a diethyl ether solution of **5a** to –35 °C yielded small yellow needles which weakly diffracted X-rays. While the data was marginal (requiring all hydrogens not bound to iron to be calculated) the refinement did afford a satisfactory solution with the molecular structure depicted in Fig. S20.† The crystallized isomer of **5a** contains a meridional chelate ligand with the formate moiety positioned proximal to the N–Me substituent. The Fe–H bond is located *trans* to the formate ligand and *cis* to the iron-carbonyl. Additional structural evidence was obtained from 2D NOESY NMR spectra (23 °C, 300 ms mixing time) which indicated a through space correlation between the Fe–H and N–CH_3_ resonances for at least one of the isomers of **5a**, though overlap between the isomers obviated assignment for this correlation to a specific isomer. Still, the NOESY NMR data indicated that the Fe–H and N–CH_3_ fragments are on the same face of the iron coordination environment for one isomer, presumably the one not identified by X-ray diffraction.^28^ Isotopic labeling of **5a** with ^13^CO afforded ^2^*J*_C–H_ coupling constants between the bound ^13^CO and Fe–H of 19.5 and 23.9 Hz for the major and minor isomers, respectively. The larger coupling constant for the minor isomer indicates a *trans* disposition of these fragments, which along with the NOESY NMR data and X-ray diffraction study is consistent with the isomers depicted in Fig. 2. This collection of data also suggests the structure determined by X-ray diffraction is the major isomer.

### CO_2_ hydrogenation activity of [^R^PN^Me^PFe] complexes

With several [^R^PN^Me^PFe] complexes in hand, the metal-hydride containing species (^iPr^PN^Me^P)Fe(H)BH_4_, **3a** and **4a** were each screened for CO_2_ hydrogenation under the conditions described for **1b**/LiOTf in Table 2. Although (^iPr^PN^Me^P)Fe(H)BH_4_ proved to be an ineffective catalyst with a TON = 52 (a conversion comparable to the reaction without iron catalyst), both **3a** and **4a** afforded very high conversions with TONs of 7660 and 6900, respectively (Table 3). The observed formate yields were in excess of the equivalents of DBU employed; however, stabilization of multiple formate ions by a single DBU *via* homoconjugation has been previously observed.15b,^29^ The dramatic improvement in catalyst performance using the *N*-methylated ligand necessitated trials at lower catalyst and higher DBU loadings to better elucidate their optimum performance. Given the comparable activity of **3a** and **4a** in preliminary experiments, the relative ease in obtaining **3a** made it a more convenient choice for exploratory catalytic trials (Table 3). Only when the catalyst loading was dropped to 0.30 μmol and the DBU/Fe ratio raised to *ca.* 40 000 did the yield of formate decrease below the concentration of DBU employed. At *ca.* 80 000 equivalents of base per iron an impressive 42 347 turnovers to formate were observed. Further enhancement of the conversion to nearly 60 000 TON was achieved by raising the LiOTf co-catalyst loading to 5/1 with base, however, at this loading not all of the LiOTf appeared to dissolve and further increasing LiOTf amounts did not enhance the conversion. The central role of the LA co-catalyst was demonstrated in a control experiment where the absence of LiOTf drops the TON to a meager 2790. Overall, this remarkably active catalyst system affords TONs more than an order of magnitude greater than any previously reported iron catalysts.

**Table 3:** CO_2_ hydrogenation catalyzed by **3a**^*a*^

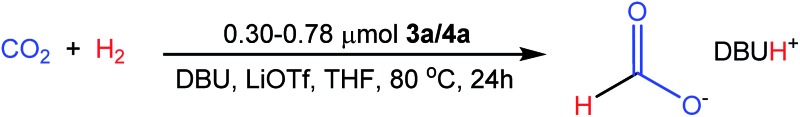					
Catalyst	[Fe] (μmol)	DBU/Fe	DBU/LiOTf	TON^*b*^	Yield^*d*^ (%)
**3a**	0.78	5000	7.5/1	7660	>99
**4a**	0.78	5000	7.5/1	6900	>99
**3a**	0.30	39 800	7.5/1	34 030	85
**3a**	0.30	79 600	7.5/1	42 350^*c*^	53
**3a**	0.30	79 600	5/1	58 990^*c*^	74
**3a**	0.30	79 600	No LiOTf	2790	4

^*a*^Reaction conditions: 69 atm of CO_2_ : H_2_ (1 : 1), 0.30 or 0.78 μmol of **3a** or **4a** in 5 mL or 10 mL THF at 80 °C for 24 hours.

^*b*^Formate production quantified by ^1^H NMR spectroscopy.

^*c*^Reported values are average of two trials.

^*d*^Reported yields are based on DBU : formate of 1 : 1.

Table 4 provides a comparison of each of the iron CO_2_ hydrogenation catalysts described herein.^30^ Each trial was conducted under 69 atm of CO_2_ : H_2_ (1 : 1) with 0.30–0.78 μmol catalyst, and 79 600 equiv. of DBU with a 7.5 : 1 loading of base to LiOTf in THF at 80 °C. Under these conditions the secondary amine containing complexes **1a** and **1b** (entries 2 and 3) improve their TONs to 6030 and 8,910, respectively, over 24 hours. Trials conducted at longer reaction times did not improve the conversion indicating the catalysts had completely deactivated after 1 day.

**Table 4:** Comparison of iron catalysts for CO_2_ hydrogenation to formate^*a*^

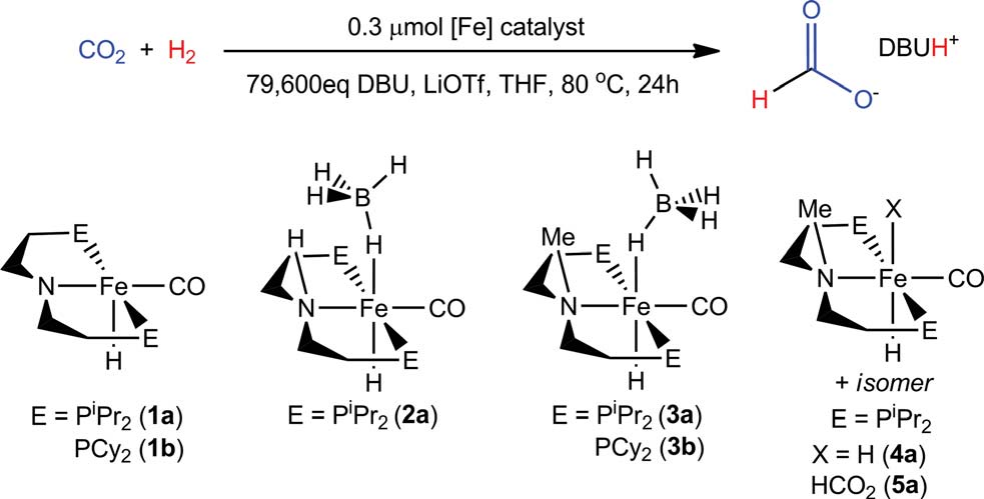					
Entry	Catalyst	DBU/LiOTf	TOF^*b*^ (1 h)	TON^*b*^ ^,^^*c*^ (24 h)	Yield^*d*^ (%)
1	**1a**	7.5/1	1290	6030	8
2	**1b**	7.5/1	1830	8910	11
3	**2a**	7.5/1	680	1500	2
4	**3a**	7.5/1	18 050	42 350	53
5	**3b**	7.5/1	20 490	46 110	58
6	**4a**	7.5/1	18 410	38 970	49
7	**5a**	7.5/1	23 190	46 130	58

^*a*^Reaction conditions: 69 atm of CO_2_ : H_2_ (1 : 1), 0.3 μmol of catalyst in 10 mL THF (*ca.* 0.01 M), 3.600 g DBU at 80 °C.

^*b*^Formate production quantified by ^1^H NMR spectroscopy. TOF was measured after first hour including a temperature equilibration period (<10 min).

^*c*^Reported values are average of two trials.

^*d*^Reported yields are based on DBU : formate of 1 : 1.

Use of the borohydride analog, (^iPr^PNP)Fe(H)CO(BH_4_) (**2a**), a feasible precatalyst for **1a**, showed dramatically lower activity (entry 3).25*c* While the secondary amine [^R^PNPFe] complexes are highly active catalysts with respect to most previously reported iron and cobalt catalysts, they pale in comparison to the tertiary amine [^R^PN^Me^PFe] systems. As noted above, **3a** delivers a remarkable 42 350 conversions to formate over 24 hours (entry 4). Much like the analogous secondary amine supported catalyst, the cyclohexyl phosphine substituted **3b** (entry 5) offers a small enhancement over the isopropyl phosphine counterpart. Use of the iron(ii) dihydride carbonyl species **4a** in place of the borohydride precursor (entries 4 and 6) afforded very similar TONs and TOFs (TOF were measured after the first hour of reaction). This is consistent with a rapid conversion of **3a** to **4a** under catalytic conditions, with both species then proceeding *via* a common mechanism for CO_2_ hydrogenation (*vide infra*).^1^ Use of **5a** as a catalyst (entry 7) gave activity comparable to **3a** and **4a**, consistent with it being an intermediate in catalysis using **3a** or **4a**. It is notable that for all the [^R^PN^Me^PFe] catalysts (entries 4–7) almost half the total conversion occurs during the first hour of the reaction. Again, no additional conversion was observed for experiments conducted for longer than 24 hours. This is indicative of highly active catalysts whose overall productivity declines significantly as the reaction proceeds.

### Mechanistic considerations of (^R^PNP)Fe(H)CO/Li^+^ catalyzed CO_2_ hydrogenation

**Scheme 1:**
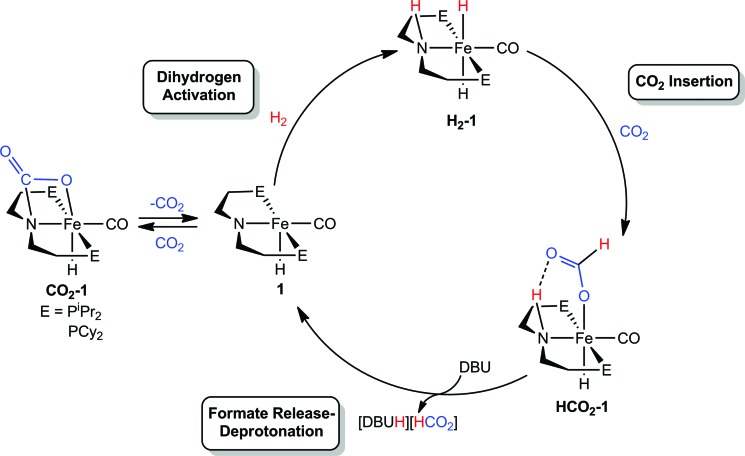
Proposed mechanism for (^R^PNP)Fe(H)CO/Li^+^ catalyzed CO_2_ hydrogenation.

We were interested in understanding the role of the LA in systems supported by both ^R^PNP and ^R^PN^Me^P ligands and the increased activity of the tertiary amine supported species. The rate influencing role of the LA co-catalyst was first explored by studying the elementary reaction steps in isolation *via* NMR spectroscopy in systems with the bifunctional ^R^PNP ligand. On the basis of our related studies,^18^ a plausible pathway for CO_2_ hydrogenation starting from **1** could proceed *via* (1) 1,2-addition of H_2_ across the Fe–N bond, followed by (2) insertion of CO_2_ into an Fe–H, and then (3) N–H deprotonation accompanied by formate extrusion to regenerate **1** (Scheme 1). Since the activities of **1a** and **1b** were comparable, –P^i^Pr_2_ substituted **1a** was selected for NMR experiments due to its simplified spectra.

**Fig. 3:**
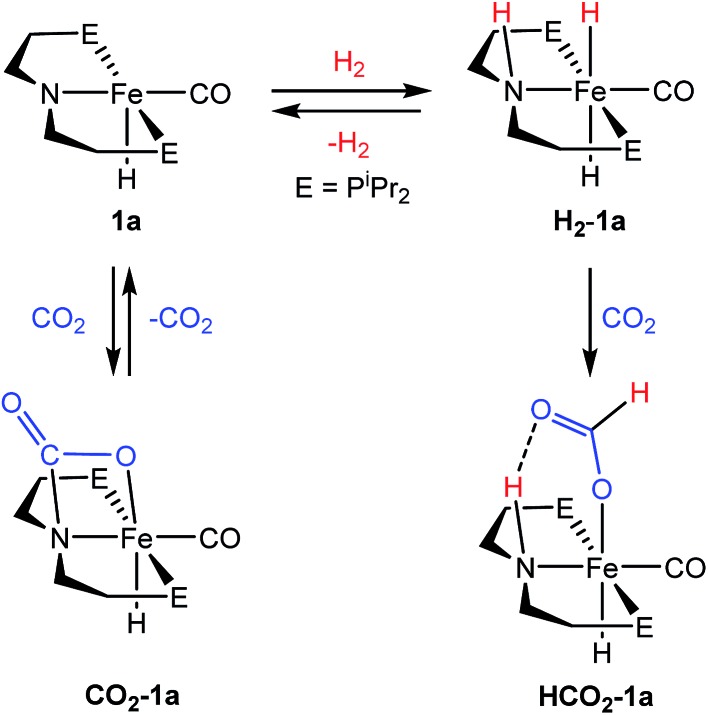
Reactions of **1a** with CO_2_ and H_2_.

The H_2_ activation reaction can be observed directly by addition of H_2_ to **1a** in THF or benzene solution, and results in near instantaneous bleaching of the dark red color and a corresponding appearance of NMR signals previously described for (^iPr^PNP)Fe(H)_2_CO (**H_2_-1a**).^18^ Though **H_2_-1a** was not isolable in the absence of an H_2_ atmosphere, CO_2_ insertion was immediately observed upon addition of 1 atm of CO_2_ to an *in situ* generated solution of **H_2_-1a** (Fig. 3). Careful examination of the NMR spectra over the first 15 minutes following CO_2_ addition revealed sufficient signals to account for the formation of two products. Two triplet Fe–H resonances were observed in the ^1^H NMR spectrum, at –25.43 and –25.83 ppm and ^31^P NMR spectra exhibited peaks at 95.40 and 93.99 ppm. The more upfield resonance in each of these pairs was assigned to **HCO_2_-1a**, which was previously prepared by addition of formic acid to **1a**.^18^ Over the course of 1 hour the resonances originating from the second product diminished with concomitant growth in the signals for **HCO_2_-1a**. The reaction sequence was repeated using ^13^CO_2_ in order to gain insight into the transient product which afforded two enhanced resonances in the ^13^C NMR spectrum at 165.37 and 174.81 ppm. ^1^H-^13^C HSQC NMR spectra showed correlation between the resonance at 174.81 ppm and the formate C–H resonance of **HCO_2_-1a** at 9.51 ppm in the ^1^H-dimension; however, no one-bond correlations were observed for the signal at 165.37 ppm. This indicated that the transient species was not simply an isomer of **HCO_2_-1a**. Instead complex **CO_2_-1a** is the formal product of CO_2_ addition across the Fe–N bond, and separate experiments show that this species may also be obtained as the sole product from the reaction of CO_2_ to **1a**. Definitive characterization of **CO_2_-1a** was established by single crystal X-ray diffraction as depicted in Fig. S21.† To the best of our knowledge, addition of CO_2_ across an Fe–NR_2_ bond has not previously been reported, but the transformation is closely related to the more commonly observed cycloaddition of CO_2_ to transition metal imides and CO_2_ insertion into transition metal amides.^31^ Though **CO_2_-1a** has limited stability to vacuum, small quantities of pure material were isolated by low temperature crystallization from pentane solution under N_2_. Notably, addition of 1 atm of H_2_ to **CO_2_-1a** affords **HCO_2_-1a** cleanly over 6–8 hours at ambient temperature with no observable intermediates. If these results are extrapolated to the catalytic conditions, this suggests that formation **CO_2_-1a** is likely of minimal consequence to CO_2_ hydrogenation, but may serve as a reversibly formed off-cycle catalytic intermediate.

Overall, the rapidity of **H_2_-1a** and **HCO_2_-1a** formation (even under temperature and pressure conditions far more mild than the catalytic reaction) suggests that extrusion of formate and/or N–H deprotonation from **HCO_2_-1a** are likely key to the rate of (^R^PNP)Fe(H)CO/Li^+^ catalyzed CO_2_ hydrogenation. This was supported by *in situ* NMR spectroscopy of a catalytic reaction under modified conditions (1 : 3 : 40 ratio of **HCO_2_-1a**:LiBF_4_ : DBU in THF under 2 atm of CO_2_/H_2_ at ambient temperature) which showed ^31^P and ^1^H NMR resonances approximate to **HCO_2_-1a** as the primary organometallic species. To better assign these resonances and gain further insight into roles of Fe, Li^+^ and DBU in this portion of the reaction, a series of stoichiometric NMR scale reactions was performed (Fig. S1†). First, samples of **HCO_2_-1a** were independently treated with 1 equiv. of DBU and LiBF_4_ in THF-*d*_8_. The sample treated with base showed no reaction, but the NMR spectra of the sample containing LA exhibited several changes indicative of an interaction between **HCO_2_-1a** and Li^+^. LiBF_4_ addition resulted in a ∼1.5 ppm upfield shift of the ^31^P NMR resonance and a corresponding downfield shift of ∼0.2 ppm for the Fe–H peak in the ^1^H NMR spectrum (Fig. S1†). A more dramatic upfield movement of the N–H resonance from 8.52 to 5.74 ppm was also observed. This change in chemical shift is consistent with a disruption of the hydrogen bonding interaction between the secondary amine and the bound formate, an interaction which has been predicted by computational analysis.^18^,^19^ While an exact structure for the Li-bound **HCO_2_-1a** complex has not been established, ^7^Li NMR spectroscopy exhibited a resonance at –2.12 ppm which is consistent with a Li–O interaction.^32^ Subsequent addition of 1 equiv. of DBU to the Li^+^/**HCO_2_-1a** complex produced only a minimal change in the ^31^P NMR spectrum, and a very modest shift of the N–H proton resonance back downfield to 6.20 ppm. This indicates that DBU does not significantly alter the hydrogen bonding interaction. Notably, no conversion to **1a** and free formate was detected by NMR spectroscopy.

**Fig. 4:**
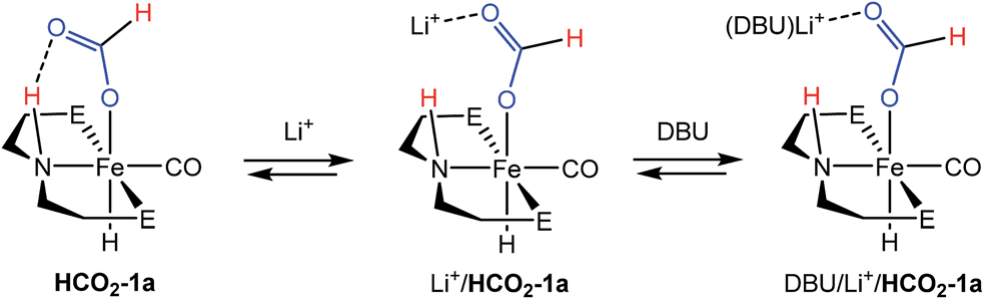
Reversible activation of **HCO_2_-1a** by Li cation and DBU.

The stoichiometric experiments suggest a three component equilibrium exists between **HCO_2_-1a**, Li^+^/**HCO_2_-1a**, and DBU/Li^+^/**HCO_2_-1a** complexes (Fig. 4). The inability to observe separate NMR resonances for these species (even at –80 °C) suggests that equilibration is rapid. The relative upfield N–H ^1^H NMR chemical shift also indicates a thermodynamic preference for the Li^+^/**HCO_2_-1a** and DBU/Li^+^/**HCO_2_-1a** complexes. However, it is important to consider that the conditions of these stoichiometric NMR experiments are far removed from the prevailing Li^+^ and DBU concentrations under catalytic conditions.

In order to better model the catalytic reaction a J. Young NMR tube was charged with DBU/LiBF_4_/**HCO_2_-1a** in a 40 : 3 : 1 ratio. Initial NMR spectra were nearly identical to those for the stoichiometric DBU/Li^+^/**HCO_2_-1a** mixture (Fig. S2†). Addition of 1.5 atm each of H_2_ and CO_2_ produced a gradual downfield shift in the N–H resonance along with the growth of a new peak near 9.30 ppm assigned to the free formate product. After 2 hours the conversion was complete and the N–H resonance remained at ∼7.80 ppm. This experiment is consistent with a reaction model where the initial resting state of the catalyst is dominated by the Li^+^ and DBU bound forms of **HCO_2_-1a**, but as DBU is consumed and the product ammonium formate reduces the available Li^+^ concentration *via* equilibration between the salts, the resting state equilibrium shifts toward the pure **HCO_2_-1a** complex.

Overall, the combination of data collected from the NMR experiments point toward the extrusion of formate from iron and/or the deprotonation of the N–H bond as the limiting steps of (^R^PNP)Fe(H)CO/Li^+^ co-catalyzed CO_2_ hydrogenation. The addition of LA appears to enhance catalysis primarily by assisting removal of the anionic formate and making the N–H fragment more available for DBU deprotonation through disruption of its hydrogen bond.

### Mechanistic considerations for [^R^PN^Me^PFe] catalysts

The mechanism of CO_2_ hydrogenation for the [^R^PN^Me^PFe] catalysts was investigated through a series of NMR spectroscopy experiments. The similar catalytic performance of the iron borohydride and dihydride catalysts, **3a** and **4a** (Table 4), as well as the synthesis of **4a** from **3a** in the presence of base, suggests that both catalysts function *via* the same mechanism. It is likely that **3a** simply serves as a precatalyst, which rapidly forms **4a** upon exposure to the high concentrations of DBU present at the initiation of the reaction. This hypothesis was supported by *in situ* monitoring using NMR spectroscopy of a catalytic reaction using **3a** or **4a** with 3 and 40 equiv. of LiBF_4_ and DBU, respectively, under 1.5 atm each of H_2_ and CO_2_ (Fig. S3–S6†). In both cases, catalytically active (^iPr^PN^Me^P)Fe(H)CO(HCO_2_) (**5a**) (see Table 4), was observed as the resting state during formate production, although some residual **3a** remained in the experiment using the iron borohydride catalyst.

The iron formate catalyst resting state for the [^R^PN^Me^PFe] systems parallels that observed for the secondary amine complexes, suggesting that formate extrusion still limits the rate of catalysis. In the case of **1a** and **1b**, our mechanistic experiments demonstrated that the Li^+^ co-catalyst assisted with this step, in part, by disrupting an intramolecular hydrogen bond between the formate and amide ligand. The role of Li^+^ was similarly probed for the [^R^PN^Me^PFe] system through stoichiometric NMR experiments. A sample of **5a** in THF-*d*_8_ was first treated with 5 equiv. of DBU under 1 atm H_2_ and monitored for 16 hours at ambient temperature, resulting in no observable formation of free formate or the iron dihydride complex **4a**. However, addition of 3 equiv. of LiBF_4_ immediately afforded full conversion to **4a** and extrusion of a formate ion (Fig. S12 and S13†). This observation is consistent with LA assistance of formate release from the iron coordination sphere, likely *via* stabilization of the anionic formate by the Li^+^ center. Further evidence for this interaction was obtained by the addition of 3 equiv. of LiBF_4_ to a THF-*d*_8_ solution of **5a**, which immediately shifted the Fe–H ^1^H NMR resonances upfield by approximately 0.5 ppm and dramatically broadened both these signals and the peaks corresponding to the formate C–H protons. A broadening and upfield shift of signals was also observed in the ^31^P NMR spectrum, suggestive of a reversible coordination of Li^+^ to **5a** (Fig. S14 and S15†).

**Scheme 2:**
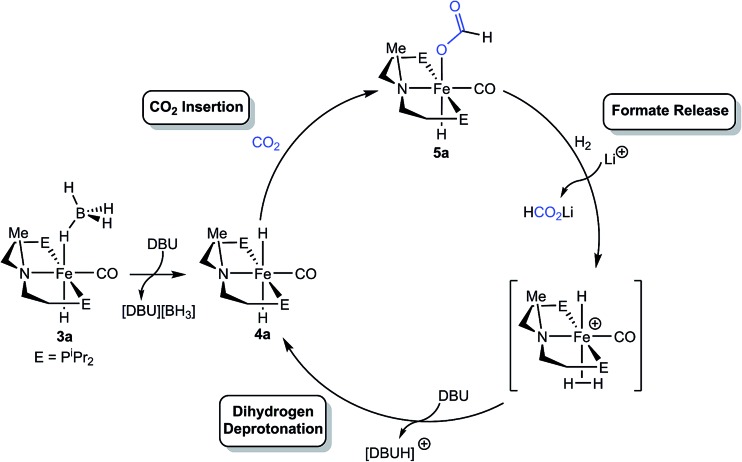
Proposed pathway for catalytic CO_2_ hydrogenation using (^iPr^PN^Me^P)Fe(H)CO(BH_4_).

The mechanistic information available suggests a pathway for [^R^PN^Me^PFe] catalyzed CO_2_ hydrogenation which shares some common features with the secondary amine containing [^R^PNPFe] catalyst (Scheme 2), including the insertion of CO_2_ into an Fe–H bond followed by rate limiting formate extrusion. Yet the absence of a bifunctional amide moiety requires distinct mechanisms for the elementary reaction steps of H_2_ activation and deprotonation by DBU. It is proposed that for the [^R^PN^Me^PFe] catalysts, Li^+^ facilitates the displacement of formate by dihydrogen to generate a transient iron(ii) dihydrogen cationic complex, which is then deprotonated by DBU to regenerate the iron(ii) dihydride species. No spectroscopic evidence for the iron(ii) dihydrogen cationic complex has been observed during catalysis, but several closely related iron complexes have been observed by others and implicated as intermediates in CO_2_ hydrogenation.3d,13a,^33^ Given the prior precedent, a formate release/H_2_ deprotonation sequence was deemed more likely than an Fe–H deprotonation/H_2_ oxidative addition pathway, which would require the intermediacy of a zerovalent iron species.

## Conclusions

The catalytic activity of a family of PNP supported iron hydrides, containing either secondary or tertiary amines was investigated. In both cases dramatic improvements in TON and TOF were observed when LA co-catalysts were present. Our best system, involving the tertiary amine supported complex (^iPr^PN^Me^P)Fe(H)CO(BH_4_), achieved approximately 60 000 turnovers, more than an order of magnitude greater than other iron catalysts and far superior to any earth abundant metal catalysts reported to date. In systems containing a secondary amine ligand, NMR spectroscopy identified the catalyst resting state as (^R^PNP)Fe(H)CO(HCO_2_) and suggested a key role for LA was disrupting a stabilizing hydrogen bond between N–H and Fe–O_2_CH moieties in this species. Mechanistic consideration of the [^R^PN^Me^PFe] catalysts afforded a model whereby (^R^PN^Me^P)Fe(H)CO(BH_4_) was activated by base to produce a (^R^PN^Me^P)Fe(H)_2_CO species which rapidly inserts CO_2_. The resulting formate complex, (^iPr^PN^Me^P)Fe(H)CO(HCO_2_), was identified as the catalytic resting state. In this case, the primary role of LA was its assistance in a formate for dihydrogen substitution which yields a transient cationic iron(ii) dihydrogen complex. Subsequent deprotonation of the dihydrogen fragment by DBU regenerates (^R^PN^Me^P)Fe(H)_2_CO. This pathway for CO_2_ hydrogenation resulted in remarkable activity, providing approximate TOFs of 20 000 h^–1^. Given that most precious and earth abundant metal catalysts are postulated to operate *via* similar mechanisms for CO_2_ hydrogenation, it is possible that the use of LA co-catalysts could dramatically enhance performance across of a range of other CO_2_ reduction systems. Such improvements in CO_2_ hydrogenation at iron may enable these or related catalyst systems to produce even higher value products, such as methanol, under optimized conditions. Targeting these CO_2_ functionalization products, as well as further elucidating the structure-reactivity relationships in the [^R^PN^Me^PFe] system are the foci of on-going efforts in our laboratories.

## Supplementary Material

Supplementary information

Crystal structure data

## References

[cit1] Dincer I. (1999). Energy Policy.

[cit2] (a) Feedstocks for the Future, ed. J. Bozell and M. K. Patel, ACS Symposium Series 921, American Chemical Society, Washington, DC, 2006.

[cit3] (d) Ziebart C. and Beller M., in Transformation and Utilization of Carbon Dioxide, ed. B. M. Bhanage and M. Arai, Springer, Berlin, 2014.

[cit4] Enthaler S., von Langermann J., Schmidt T. (2010). Energy Environ. Sci..

[cit5] Yasaka Y., Wakai C., Matubayasi N., Nakahara M. (2010). J. Phys. Chem. A.

[cit6] Shintani R., Nozaki K. (2013). Organometallics.

[cit7] Laurenczy G., Joó F., Nádasdi L. (2000). Inorg. Chem..

[cit8] (b) Leitner W., Dinjus E. and Gassner F., in Aqueous-Phase Organometallic Catalysis, Concepts and Applications, ed. B. Cornils and W. A. Herrmann, Wiley-VCH, Weinheim, 1998, p. 486.

[cit9] Joo F., Joo F., Nadasdi L., Elek J., Laurenczy G., Nadasdi L. (1999). Chem. Commun..

[cit10] Tanaka R., Yamashita M., Nozaki K. (2009). J. Am. Chem. Soc..

[cit11] Inoue Y., Izumida H., Sasaki Y., Hashimoto H. (1976). Chem. Lett..

[cit12] Hansen J. B., Handbook of Heterogeneous Catalysis, ed. G. Ertl, H. Knötzinger and J. Weitkamp, VCH, 1997, vol. 4.

[cit13] Fong H., Peters J. C. (2015). Inorg. Chem..

[cit14] Badiei Y. M., Wang W.-H., Hull J. F., Szalda D. J., Muckerman J. T., Himeda Y., Fujita E. (2013). Inorg. Chem..

[cit15] Jeletic M. S., Mock M. T., Appel A. M., Linehan J. C. (2013). J. Am. Chem. Soc..

[cit16] Federsel C., Boddien A., Jackstell R., Jennerjahn R., Dyson P. J., Scopelliti R., Laurenczy G., Beller M. (2010). Angew. Chem., Int. Ed..

[cit17] Langer R., Diskin-Posner Y., Leitus G., Shimon L. J. W., Ben-David Y., Milstein D. (2011). Angew.
Chem., Int. Ed..

[cit18] Bielinski E. A., Lagaditis P. O., Zhang Y., Mercado B. Q., Würtele C., Bernskoetter W. H., Hazari N., Schneider S. (2014). J. Am. Chem. Soc..

[cit19] Bielinski E. A., Förster M., Zhang Y., Bernskoetter W. H., Hazari N., Holthausen M. C. (2015). ACS Catal..

[cit20] To the best of our knowledge the use of Lewis acids to improve CO_2_ hydrogenation to formate is conceptually novel. Previously Leitner and coworkers have used Brønsted acids to improve TONs in the hydrogenation of CO_2_ to methanol. See: Wesselbaum S. von Stein T. Klankermayer J. Leitner W., Angew. Chem., Int. Ed., 2012, 51 , 7499 .

[cit21] Fan L., Foxman B. M., Ozerov O. V. (2004). Organometallics.

[cit22] Chakraborty S., Brennessel W. W., Jones W. D. (2014). J. Am. Chem. Soc..

[cit23] Bornschein C., Werkmeister S., Wendt B., Jiao H., Alberico E., Baumann W., Junge H., Junge K., Beller M. (2014). Nat. Commun..

[cit24] Addison A. W., Rao T. N., Reedijk J., van Rijn J., Verschoor G. C. (1984). J. Chem. Soc., Dalton Trans..

[cit25] Marks T. J., Kolb J. R. (1977). Chem. Rev..

[cit26] Similar spectra have been observed for the cyclohexyl congener, **3b**, but no attempt has been made to isolate (^Cy^PN^Me^P)Fe(H)_2_CO

[cit27] The metrical parameters of each polymorph of **4a** are quite similar. The cifs for both polymorphs are provided in the ESI.

[cit28] No exchange correlations between isomers of **5a** were observed under these conditions

[cit29] Munshi P., Main A. D., Linehan J. C., Tai C.-C., Jessop P. G. (2002). J. Am. Chem. Soc..

[cit30] In the absence of iron catalyst, only 0.1% conversion to formate was observed under these conditions.

[cit31] Chakraborty S., Blacque O., Berke H. (2015). Dalton Trans..

[cit32] Reich H. J., Borst J. P., Dykstra R. R., Green P. D. (1993). J. Am. Chem. Soc..

[cit33] Gilbertson J. D., Szymczak N. K., Tyler D. R. (2004). Inorg. Chem..

