# Supplementary material for: Iron catalyzed CO2 hydrogenation to formate enhanced by Lewis acid co-catalysts
Source: Chem Sci. 2015 May 28;6(7):4291–9. doi: 10.1039/c5sc01467k (PMC5707511; doi:10.1039/c5sc01467k)
Supplement: Supplementary file 1 [file SC-006-C5SC01467K-s001.pdf]

Electronic Supplementary Material (ESI) for Chemical Science.
This journal is © The Royal Society of Chemistry 2015

Supporting Information: Iron Catalyzed CO_2 Hydrogenation to Formate Enhanced by Lewis Acid Co-Catalysts

Yuanyuan Zhang,[a] Alex D. MacIntosh,[a] Janice L. Wong,[b] Elizabeth A. Bielinski,[b] Paul G. Williard,[a] Brandon Q. Mercado[b], Nilay Hazari[b,*] and Wesley H. Bernskoetter[a,*]

[a]*The Department of Chemistry, Brown University, Providence, RI 02912*

[b]*The Department of Chemistry, Yale University, New Haven, CT 06520*

e-mail: nilay.hazari@yale.edu; wb36@brown.edu

Table of Contents:

General Experimental Methods and Characterizing Data S2

Tables of Base Screening and Lewis Acid Loading S8

Experimental Descriptions and NMR Spectral Data S9

X-ray Crystallography S22

References S25

General Experimental Methods and Characterizing Data

General Considerations. All manipulations were carried out using standard vacuum, Schlenk, cannula, or glovebox techniques. Hydrogen and carbon dioxide were purchased from Corp Brothers and used as received. Compounds **1a**, **1b**, **HCO_2-1a** and **2a** were prepared according to literature procedures.[1] Compounds ($^{iPr}PN^{Me}P$)Fe(H)BH_4, **3a** and **4a** were prepared in a manner similar to that described by Beller and coworkers, but with the slight modifications described below. The ligand MeN{$CH_2CH_2(PCy_2)$}$_2$ was also prepared by analogy to a previous report, only substituting $LiPCy_2$ for LiP^iPr_2.[1a,1b] All other chemicals were purchased from Aldrich, Fisher, VWR, Strem, or Cambridge Isotope Laboratories. Non-volatile solids, surely including Cs_2CO_3, were dried under vacuum at 50 °C overnight. 1,8-diazabicycloundec-7-ene (DBU) was dried over CaH_2 and distilled prior to use. Solvents were dried and deoxygenated using literature procedures.[2] ^1H, ^{13}C and ^{31}P NMR spectra were recorded on Bruker DRX 400 MHz, Avance 300 and 600 MHz spectrometers at ambient temperature, unless otherwise noted. ^1H and ^{13}C chemical shifts are referenced to residual solvent signals; ^{31}P and ^7Li chemical shifts are referenced to the external standards of H_3PO_4 and LiCl, respectively. Probe temperatures were calibrated using ethylene glycol and methanol as previously described.[3] It should be noted that some NMR experiments employed a J. Young NMR tube with up to 3 atm of total pressure and appropriate safety precautions need to be applied if reproducing these experiments. IR spectra were recorded on Jasco 4100 FTIR and Mettler Toledo React IR spectrometers. X-ray crystallographic data were collected on a Bruker D8 QUEST diffractometer. Samples were collected in inert oil and quickly transferred to a cold gas stream. The structures were solved from direct methods and Fourier syntheses and refined by full-matrix least-squares procedures with anisotropic thermal parameters for all non-hydrogen atoms. Crystallographic calculations

were carried out using SHELXTL. The crystal data has been deposited in the CCDC under the following reference numbers: $(^{iPr}PN^{Me}P)FeCl_2$ (1061151), $(^{iPr}PN^{Me}P)Fe(H)BH_4$ (1061152), **3a** (1061153), **4a** (1061154 and 1061155), **5a** (1061156), and **CO_2-1a** (1061157). High pressure catalytic CO_2 hydrogenation reactions were performed using a Parr 5500 series compact reactor with glass insert. Elemental analyses were performed at Atlantic Microlab, Inc., in Norcross, GA or Robertson Microlit Laboratory in Ledgewood, NJ.

Preparation of CO_2-1a. A J. Young NMR tube was charged with $(^{iPr}PNP)Fe(H)CO$ (**1a**) (20 mg, 0.051 mmol) in 2 mL pentane. The sample was frozen at -198 °C, degassed, and 1 atm of carbon dioxide admitted to the tube. Upon warming to ambient temperature, the color immediately changed from magenta to yellow. After 5 minutes, the excess carbon dioxide gas was removed *in vacuo*, leaving approximately 1 mL of the product solution in the tube. The mixture was then decanted, concentrated to approximately 0.5 mL, and cooled to -35 °C overnight to afford 19 mg (85%) of **CO_2-1a** as yellow-orange crystals. ^1H NMR (C_6D_6): δ -25.44 (t, 53.9 Hz, 1H, Fe-*H*), 0.80 (m, 6H, $CH(CH_3)_2$), 1.02 (m, 6H, $CH(CH_3)_2$), 1.09 (m, 6H, $CH(CH_3)_2$), 1.19 (m, 6H, $CH(CH_3)_2$), 1.36 (m, 2H, PCH_2), 1.71 (m, 2H, PCH_2), 1.77 (m, 2H, $CH(CH_3)_2$), 1.98 (m, 2H, NCH_2), 2.13 (m, 2H, $CH(CH_3)_2$), 3.29 (m, 2H, NCH_2). $^{13}C\{^1H\}$ NMR (C_6D_6): δ 18.79, 19.18, 19.24, 19.29, 23.71, 26.21, 26.77, 56.06 (t, 4.0 Hz), 165.37 (CO_2), 236.65 (Fe-*C*O). $^{31}P\{^1H\}$ NMR (C_6D_6): δ 96.03 (s). IR (KBr): 1891 (C≡O), 1727(O=C).

Preparation of $(^{iPr}PN^{Me}P)FeCl_2$. $FeCl_2$ (167 mg, 1.32 mmol) was added to a 40 mL THF solution of $MeN\{CH_2CH_2P(^iPr)_2\}_2$ (421 mg, 1.32 mmol) at ambient temperature and stirred for 11 hours. The resulting yellow solution was then filtered, the filtrate concentrated to 10 mL and layered with approximately 5 mL pentane. Cooling overnight at -35 °C afforded 378 mg (64%) of $(^{iPr}PN^{Me}P)FeCl_2$ as a colorless crystalline solid. Magnetic susceptibility: 4.72 μB (Evans'

Method). ^{1}H NMR (THF-d_8): δ 73.9, 63.1, 46.8, 4.36, 2.98. Anal. Calcd. for $C_{17}H_{39}Cl_2FeNP_2$: C, 45.76%; H, 8.81%, N, 3.14%. Found: C, 45.57%, 8.54%, 3.02%.

Preparation of ($^{Cy}PN^{Me}P$)$FeCl_2$. This compound was prepared in a manner identical to ($^{iPr}PN^{Me}P$)$FeCl_2$ using MeN{$CH_2CH_2P(Cy)_2$}$_2$ (210 mg, 0.438 mmol) and $FeCl_2$ (52 mg, 0.410 mmol) which afforded 244 mg (98%) of a colorless powder. Magnetic susceptibility: 4.96 μB (Evans' Method). ^{1}H NMR (C_6D_6): δ 69.7, 55.6, 6.57, 4.86, 0.81, -0.13, -2.48, -5.18. Anal. Calcd. for $C_{29}H_{55}Cl_2FeNP_2$: C, 57.43%; H, 9.14%, N, 2.31%. Found: C, 57.52%, 8.86%, 2.31%.

Preparation of ($^{Cy}PN^{Me}P$)Fe(H)(BH_4). ($^{Cy}PN^{Me}P$)$FeCl_2$ (159 mg, 0.262 mmol) was dissolved in a solvent mixture containing 5 mL acetonitrile and 15 mL ethanol to provide a red-purple solution. Upon cooling the solution in an in ice-water bath, excess $NaBH_4$ (117 mg, 3.14 mmol) was added in one portion. Gas evolution was immediately observed and the solution turned brown. After stirring at ambient temperature for one hour, the brown reaction mixture was placed under reduced pressure to remove the volatiles. The residue was triturated with pentane (3×5 mL), extracted with 15 mL of pentane, and the dark brown solution concentrated. Cooling to -35 °C overnight afforded 101 mg (70%) of ($^{Cy}PN^{Me}P$)Fe(H)(BH_4) as brown crystals. The same procedure was also used to obtain ($^{iPr}PN^{Me}P$)Fe(H)(BH_4) which was identified by comparison to previously reported NMR spectra. Characterization data for ($^{Cy}PN^{Me}P$)Fe(H)(BH_4): Anal. Calcd. for $C_{29}H_{60}BFeNP_2$: C, 63.17%; H,10.97%, N 2.54%. Found: C, 63.39; H, 11.00%; N, 2.61%. ^{1}H NMR (C_6D_6): δ -35.95 (br, 1H, Fe-*H*-B), -23.00 (t, 53.1 Hz, 1H, Fe-*H*), -11.95 (br, 1H, Fe-*H*-B), 1.22-1.55 (m, 22H, P*Cy*$_2$), 1.58 (s, 3H, NC*H*$_3$), 1.59-1.69 (m, 6H, P*Cy*$_2$), 1.71-1.84 (m, 8H, P*Cy*$_2$), 1.90-1.92 (m, 4H, P*Cy*$_2$), 1.92-1.95 (m, 2H, C*H*$_2$), 2.30 (m, 2H, C*H*$_2$), 2.31 (m, 2H, P*Cy*$_2$), 2.51 (m, 2H, C*H*$_2$), 2.75 (m, 2H, P *Cy*$_2$), 3.27 (m, 2H, C*H*$_2$), 4.36 (b, 2H, B*H*$_2$). ^{13}C {^{1}H} NMR

(C_6D_6): δ 27.77, 27.80, 27.84, 27.91, 28.14, 28.21, 28.46, 28.49, 29.21, 29.68, 30.82, 36.31 (P*Cy*$_2$), 29.71, 66.58 (*C*H_2) 50.61 (N*C*H_3). $^{31}P\{^1H\}$ NMR (C_6D_6): δ 87.2 (s, PCy_2).

Preparation of ($^{Cy}PN^{Me}P$)Fe(H)CO(BH_4) (3b). A 25 mL heavy walled glass vessel was charged with ($^{Cy}PN^{Me}P$)Fe(H)BH_4 (39 mg, 0.071 mmol) in 8 mL pentane. The sample was frozen at -198 °C, degassed, and 1 atm of carbon monoxide admitted to the vessel. Upon warming to ambient temperature the color changed from brown to yellow and within 30 minutes yellow crystalline solids precipitated. Most of the volatiles were removed quickly under vacuum, leaving approximately 3 mL pentane in the vessel. Under N_2 the mixture was decanted and the yellow crystalline residue was washed with 2×3 mL of pentane. Following air-drying, the yellow solid was briefly dried under vacuum (less than 5 minutes) to afford 38 mg (92%) of **3b**. The same procedure was also used to obtain ($^{iPr}PN^{Me}P$)Fe(H)CO(BH_4) (**3a**) which was identified by comparison to previously reported NMR spectra.[1a,1b] Characterization data for ($^{Cy}PN^{Me}P$)Fe(H)CO(BH_4) (**3b**): Anal. Calcd. for $C_{30}H_{60}BFeNOP_2$: C, 62.19%; H, 10.44%, N, 2.42%. Found: C, 61.98; H, 10.18%; N, 2.24%. ^1H NMR (C_6D_6): δ -19.60 (t, 52.8 Hz, 1H, Fe-*H*), -2.54 (br, 4H, B*H*$_4$), 1.21-1.25 (m, 8H, P*Cy*$_2$), 1.40-1.47 (m, 4H, P*Cy*$_2$), 1.55-1.99 (m, 32H, C*H*$_2$ & P*Cy*$_2$), 2.01 (s, 3H, N*H*$_3$), 2.02 (m, 2H, P*Cy*$_2$), 2.18 (m, 2H, C*H*$_2$), 2.74 (m, 2H, P*Cy*$_2$), 3.07 (m, 2H, C*H*$_2$). $^{13}C\{^1H\}$ NMR (C_6D_6): δ 27.26, 27.30, 27.35, 27.65, 27.91, 28.21, 28.25, 28.94, 29.10, 31.48, 37.13, 41.81 (P*Cy*$_2$), 50.94 (N*C*H_3), 31.06, 66.00 (*C*H_2), CO resonance not located. $^{31}P\{^1H\}$ NMR (C_6D_6): δ 85.40 (s, PCy_2).

Preparation of ($^{iPr}PN^{Me}P$)Fe(H)$_2$CO (**4a**). A 20 mL scintillation vial was charged with ($^{iPr}PN^{Me}P$)Fe(H)CO(BH_4) (**3a**) (20 mg, 0.048 mmol) in 5 mL of THF. Excess NEt_3 (approximately 500 μL) was added to the solution and stirred for 20 minutes. The volatiles were then removed under vacuum and the residue extracted with 2 mL of pentane. Cooling the

solution to -35 °C overnight afforded 16 mg (89%) of **4a** as yellow needles. The compound was characterized by comparison to previously reported NMR spectra.[1a,1b]

Preparation of ($^{iPr}PN^{Me}P$)Fe(H)CO(HCO_2) (5a). A J. Young NMR tube was charged with ($^{iPr}PN^{Me}P$)Fe$(H)_2$CO (**4a**) (30 mg, 0.074 mmol) in 1 mL of C_6D_6. The sample was frozen at -198 °C, degassed, and 1 atm of carbon dioxide admitted to the tube. The tube was warmed to ambient temperature and allowed to sit for approximately 20 minutes. The volatiles were removed *in vacuo*, leaving a yellow residue. The residue was extracted with pentane (3×2 mL), concentrated to approximately 1 mL, and cooled to -35 °C overnight to afford 30 mg (90%) of **5a** as yellow crystals. Anal. Calcd. for $C_{19}H_{41}FeNO_3P_2$: C, 50.79%; H, 9.20%; N, 3.12%. Found: C, 50.72%; H, 8.47%; N, 3.04%. *Major Isomer:* ^{1}H NMR (C_6D_6): δ -23.89 (t, 52Hz, 1H, Fe-*H*), 0.90 (m, 6H, CH(CH_3)$_2$), 1.13 (m, 6H, CH(CH_3)$_2$), 1.18 (m, 6H, CH(CH_3)$_2$), 1.45 (m, 6H, CH(CH_3)$_2$), 1.51-1.58 (m, 4H, C*H*(CH_3)$_2$), 1.83 (s, 3H, CH_3), 1.96-2.10 (m, 6H), 2.31 (m, 2H), 9.22 (s, 1H, *H*CO_2). ^{13}C{^{1}H} NMR (C_6D_6): δ 18.24, 19.35, 20.03, 20.74, 25.95, 26.38, 26.86, 45.32, 65.51, 169.26 (H*C*O_2), 223.46 (*C*O). ^{31}P{^{1}H} NMR (C_6D_6): δ 87.23 (s). *Selected data for minor isomer:* ^{1}H NMR (C_6D_6): δ -24.03 (t, 51.4 Hz, 1H, Fe-*H*), 9.11 (s, 1H, *H*CO_2). ^{13}C{^{1}H} NMR (C_6D_6): δ 222.64 (*C*O). ^{31}P{^{1}H} NMR (C_6D_6): δ 89.87 (s). Sample spectra containing all peaks for the major and minor isomers are provided later in the Supporting Information.

General Methods for Catalytic CO_2 Hydrogenation Studies. In a glovebox, a 50 mL glass reactor liner was charged with catalyst as a stock solution in THF (*ca.* 0.02 M), a corresponding amount of DBU, Lewis acid and 5 mL to 10 mL THF. The cylinder liner was placed into the Parr reactor and the vessel sealed. The reactor was removed from the dry box and pressurized with 69 atm of a 1:1 CO_2:H_2 mixture at ambient temperature. The reactor was then heated and stirred at 80 °C for the indicated time. The reaction was stopped by removal from the heat source and

venting of the gases. The contents of the reactor were then quickly transferred to a 100 mL round bottom flask with assistance of D_2O to dissolve the solid products. Then all of the volatiles were removed under reduced pressure. The residue was dissolved in D_2O, and 10 to 400 μL DMF was added as an internal standard for quantification of the formate product by ^{1}H NMR spectroscopy. Fairly large amounts of D_2O may be required to completely dissolve the generated formate.

Table S1. Screening of bases for CO_2 hydrogenation catalyzed by 1b.[a]

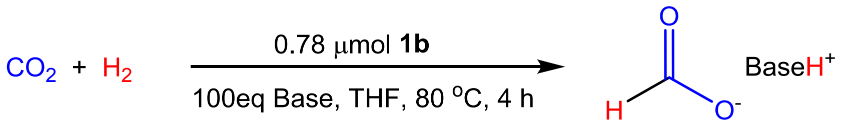

Base[d]	TON[b]	Yield (%)[e]
KHMDS[c]	4	4
KO^{t}Bu	20	20
Cs_2CO_3	60	60
NEt_3	3	3
Pyridine	1	1
DABCO[c]	50	50
DBU[c]	80	90

[a]Reaction conditions: 69 atm of CO_2:H_2 (1:1), 0.78 μmol of **1b** in 5 mL THF (*ca.* 0.015 M) at 80 °C. [b]Formate production quantified by ^{1}H NMR spectroscopy. [c]KHMDS is potassium bis(trimethylsilyl)amide, DABCO is 1,4-diazabicyclo[2.2.2]octane and DBU is 1,8-Diazabicyclo[5.4.0]undec-7-ene. [d]Et_3N and pyridine were dried over CaH_2 and molecular sieves respectively and distilled under reduced pressure. DABCO were purified by sublimation (50 °C, 0.02 mbar). DBU was dried over CaH_2 and distilled prior to use. Cs_2CO_3 was dried under vacuum at 50 °C overnight. [e]Reported yield is based on base (base/foremate = 1:1).

Table S2. Lewis acid optimization for CO_2 hydrogenation catalyzed by 1b.[a]

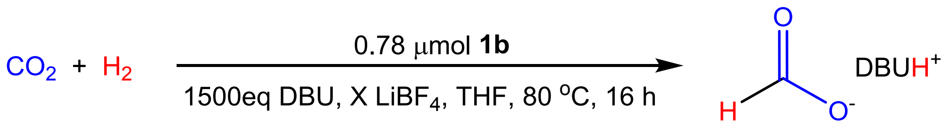

DBU/$LiBF_4$	TON[b]	Yield (%)[d]
no $LiBF_4$	431[c]	28
150/1	541	36
30/1	693	46
20/1	784	52
15/1	930	62
6/1	1190	79
5/1	1210	80
2/1	1230[c]	82

[a]Reaction conditions: 69 atm of CO_2:H_2 (1:1), 0.78 μmol of **1b** in 5 mL THF (*ca.* 0.015 M), 180 mg DBU at 80 °C. [b]Formate production quantified by ^{1}H NMR spectroscopy. [c]Reported values are the average of three trials. [d]Reported yield is based on base (base/formate = 1:1)

Treatment of HCO_2-1a with $LiBF_4$ and DBU. A J. Young NMR tube was charged with 3 mg of **HCO_2-1a** in approximately 1 mL of THF-*d_8*. Then, 3 eq of $LiBF_4$ was added under an N_2 atmosphere. After 20 minutes, approximately 40 eq of DBU was also added. The reaction was monitored by ^{31}P and ^{1}H NMR spectroscopy over the course of 16 hours.

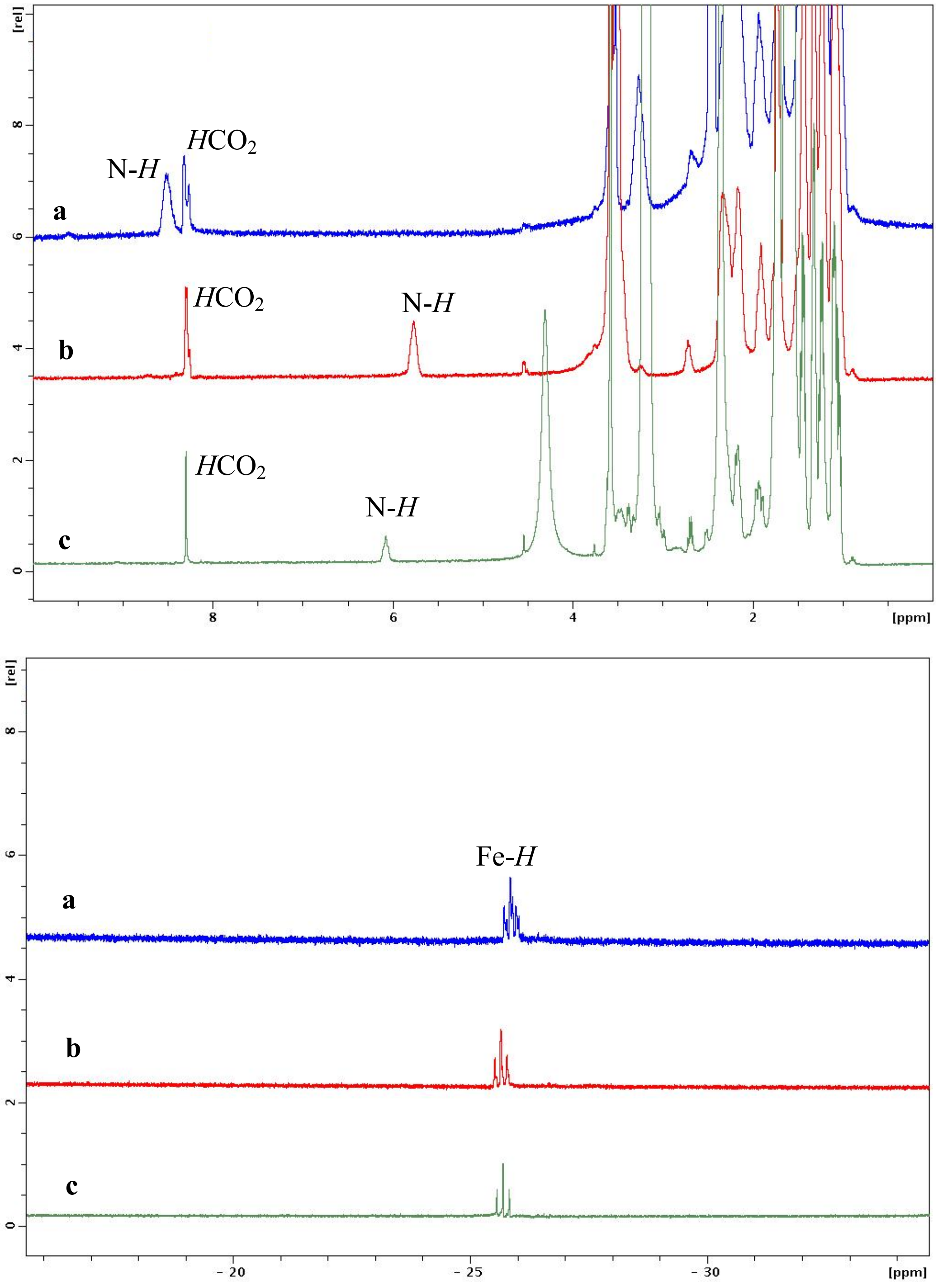

Figure S1. (a) ^{1}H NMR spectra of **HCO_2-1a**, (b) after addition of 3 eq $LiBF_4$, (c) after addition of 40 eq DBU.

NMR scale catalytic CO_2 hydrogenation reaction with HCO_2-1a. A J. Young NMR tube was charged with 5 mg of **HCO_2-1a** in approximately 1 mL of THF-*d*$_8$. Then 3 eq of $LiBF_4$ and 40 eq of DBU were added to the sample. The sample was frozen at -198 °C, degassed, and 1 atm each of carbon dioxide and dihydrogen were admitted to the tube. The sample was warmed to ambient temperature and monitored by ^{31}P and ^{1}H NMR spectroscopy.

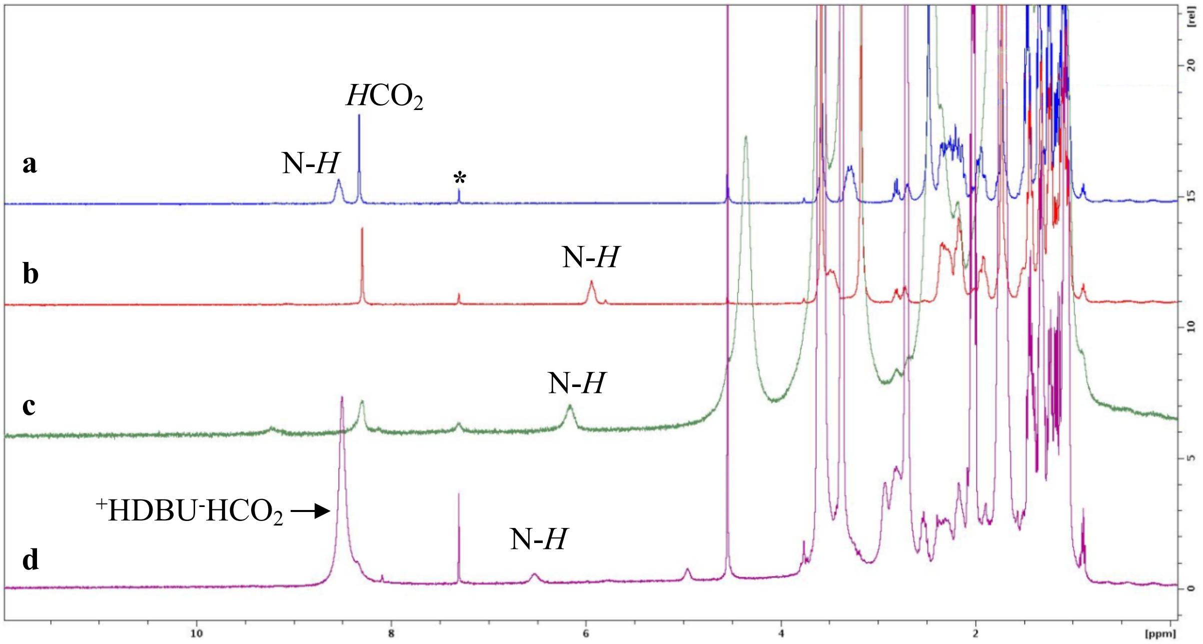

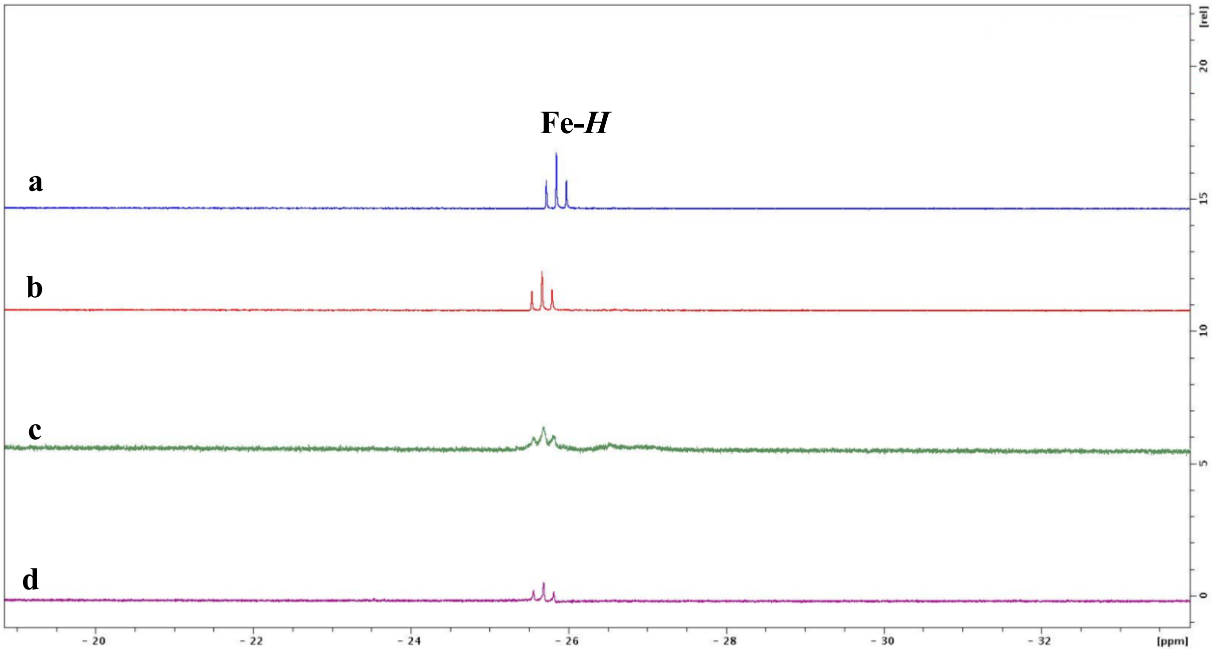

Figure S2. (a) ^{1}H NMR spectra of **HCO_2-1a**, (b) after addition of 3 eq $LiBF_4$, (c) after addition of 40 eq DBU, (d) 16 h after addition of 1 atm CO_2/H_2. *denotes C_6H_6 from glovebox atmosphere.

NMR scale catalytic CO_2 hydrogenation reaction with 3a. A J. Young NMR tube was charged with 6 mg of **3a** in approximately 1 mL of THF-*d*$_8$. Then, 3 eq of $LiBF_4$ and 40 eq of DBU were added to the sample. The sample was frozen at -198 °C, degassed, and 1 atm each of carbon dioxide and dihydrogen were admitted to the tube. The sample was warmed to ambient temperature and monitored by ^{31}P and ^{1}H NMR spectroscopy.

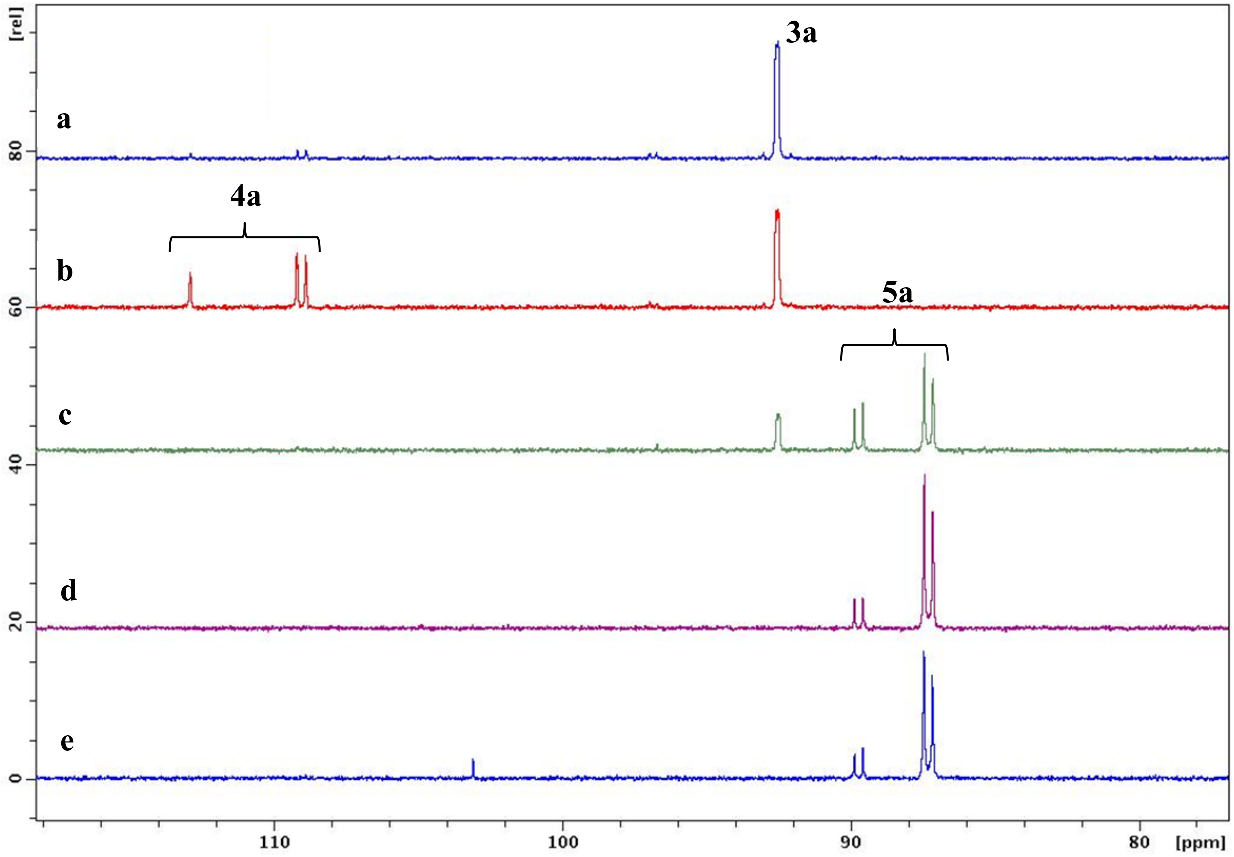

Figure S3. (a) ^{31}P NMR spectra of **3a**, (b) after addition of 3 eq $LiBF_4$, (c) after addition of 40 eq DBU, (d) 1.5 h after addition of 1 atm CO_2/H_2, (e) 16 h after addition of 1 atm CO_2/H_2.

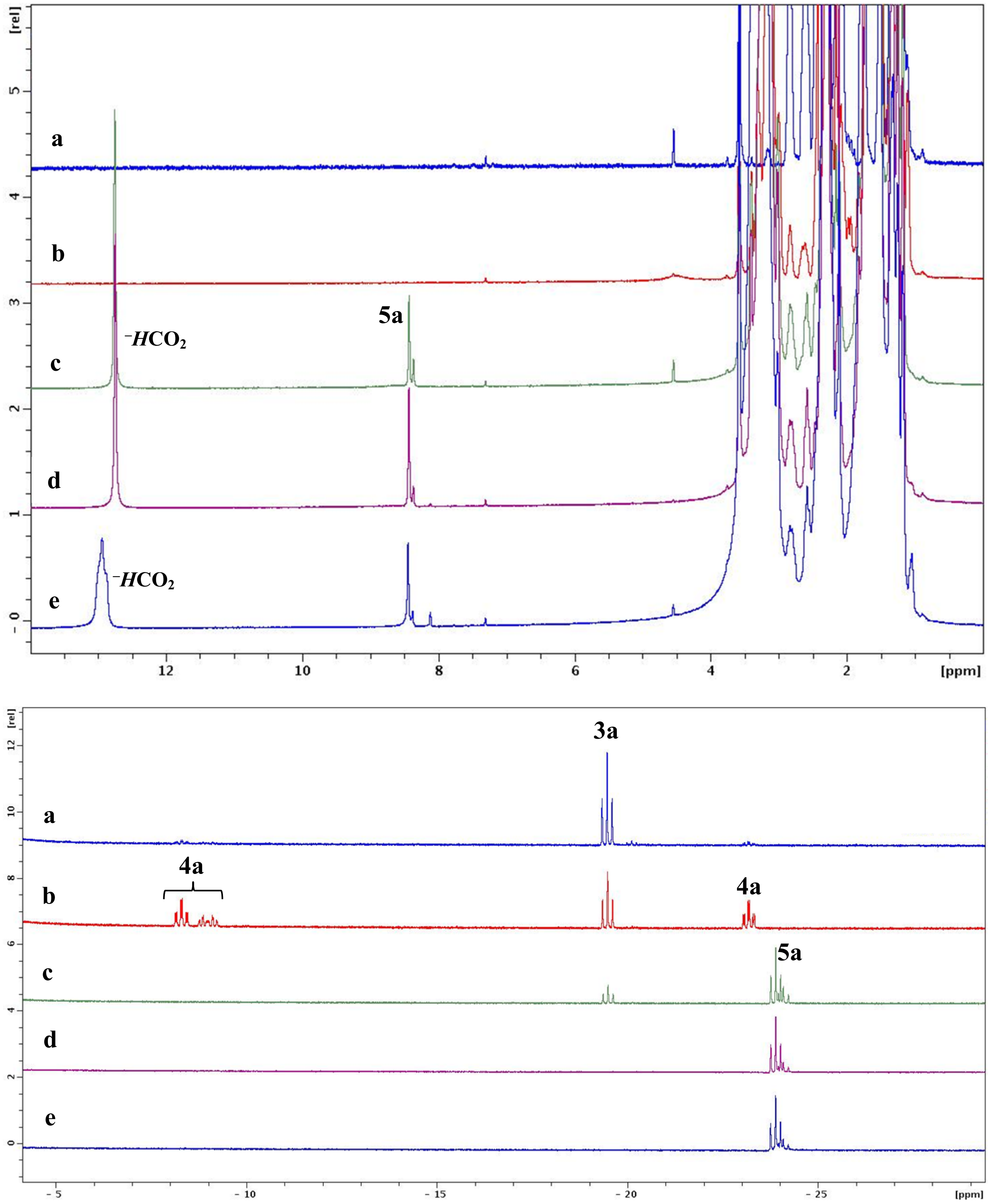

Figure S4. (a) ^{1}H NMR spectra of **3a**, (b) after addition of 3 eq $LiBF_4$, (c) after addition of 40 eq DBU, (d) 1.5 h after addition of 1 atm CO_2/H_2, (e) 16 h after addition of 1 atm CO_2/H_2. Free formate ($^{+}HDBU\ ^{-}HCO_2$) is seen upon CO_2/H_2 addition (d and e).

NMR scale catalytic CO_2 hydrogenation reaction with 4a. A J. Young NMR tube was charged with 6 mg of **4a** in approximately 1 mL of THF-d_8. Then 3 eq of $LiBF_4$ and 40 eq of DBU were added to the sample. The sample was frozen at -198 °C, degassed, and 1 atm each of carbon dioxide and dihydrogen were admitted to the tube. The sample was warmed to ambient temperature and monitored by ^{31}P and ^{1}H NMR spectroscopy over the course of 16 hours.

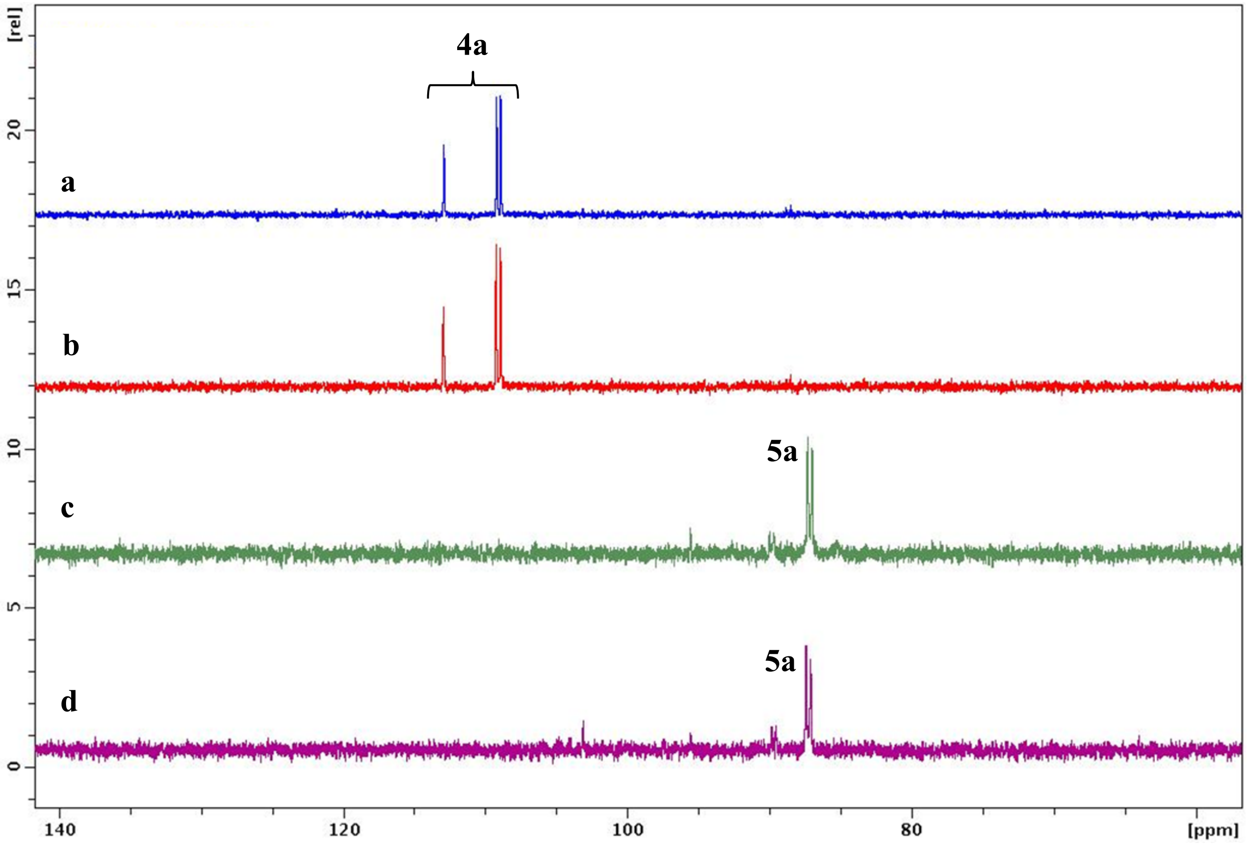

Figure S5. (a) ^{31}P NMR spectra of **4a**, (b) after addition of 3 eq $LiBF_4$ and 40 eq DBU, (c) 1.5 h after addition of 1 atm CO_2/H_2, (d) 16 h after addition of 1 atm CO_2/H_2.

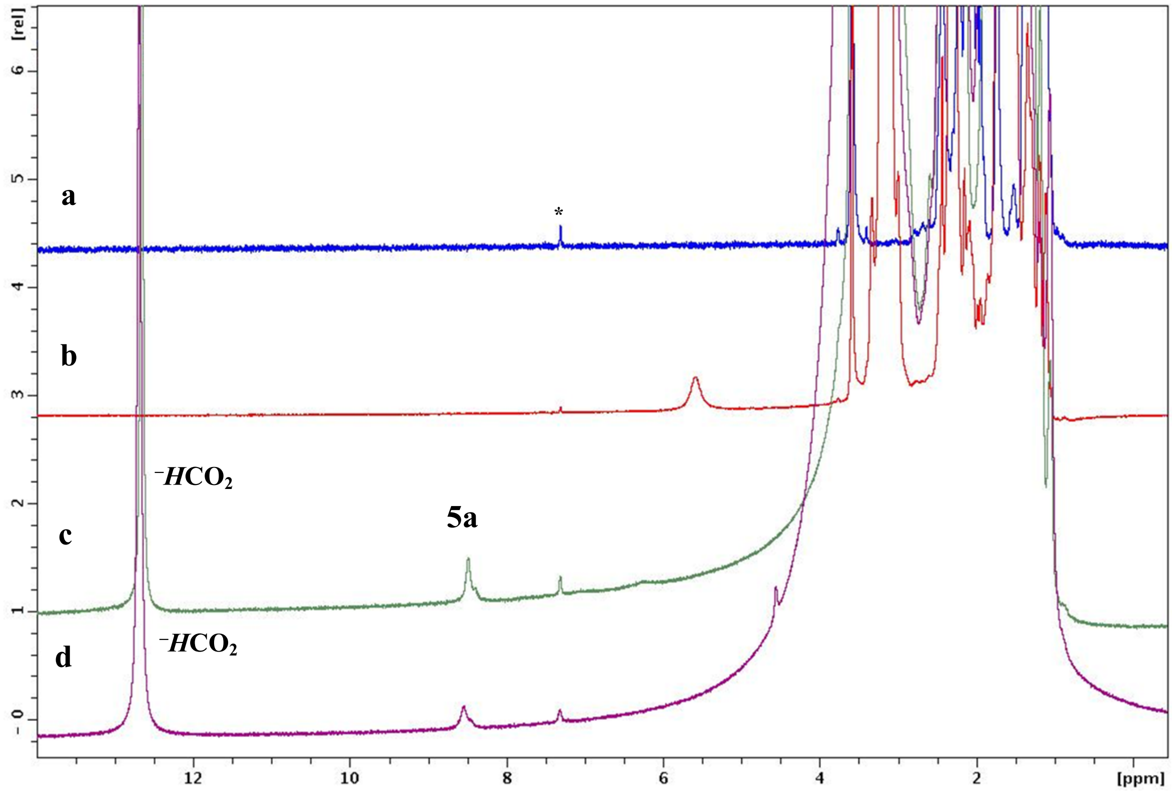

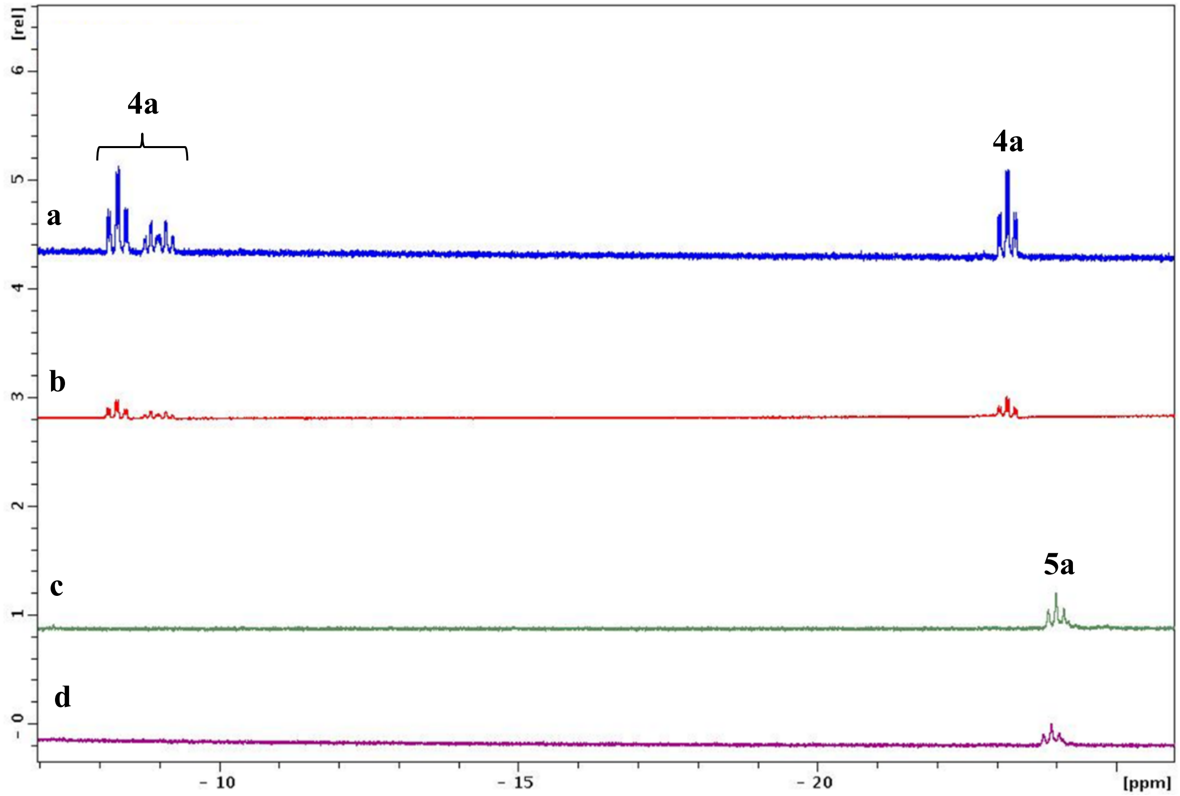

Figure S6. (a) ^{1}H NMR spectra of **4a**, (b) after addition of 3 eq $LiBF_4$ and 40 eq DBU, (c) 1.5 h after addition of 1 atm CO_2/H_2, (d) 16 h after addition of 1 atm CO_2/H_2.

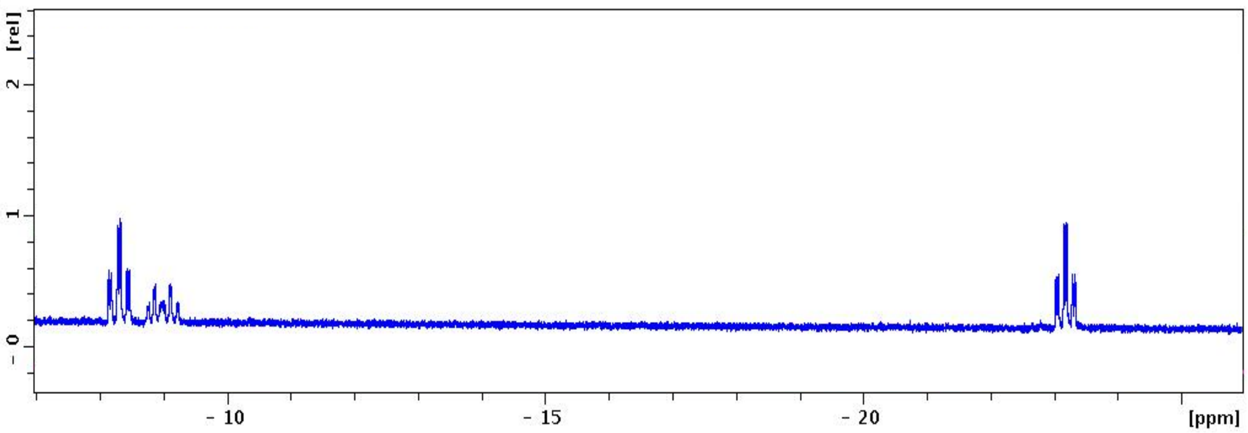

Figure S7. Partial ^{1}H NMR spectrum for **4a** showing the *cis*- and *trans*-isomers isomers in C_6D_6.

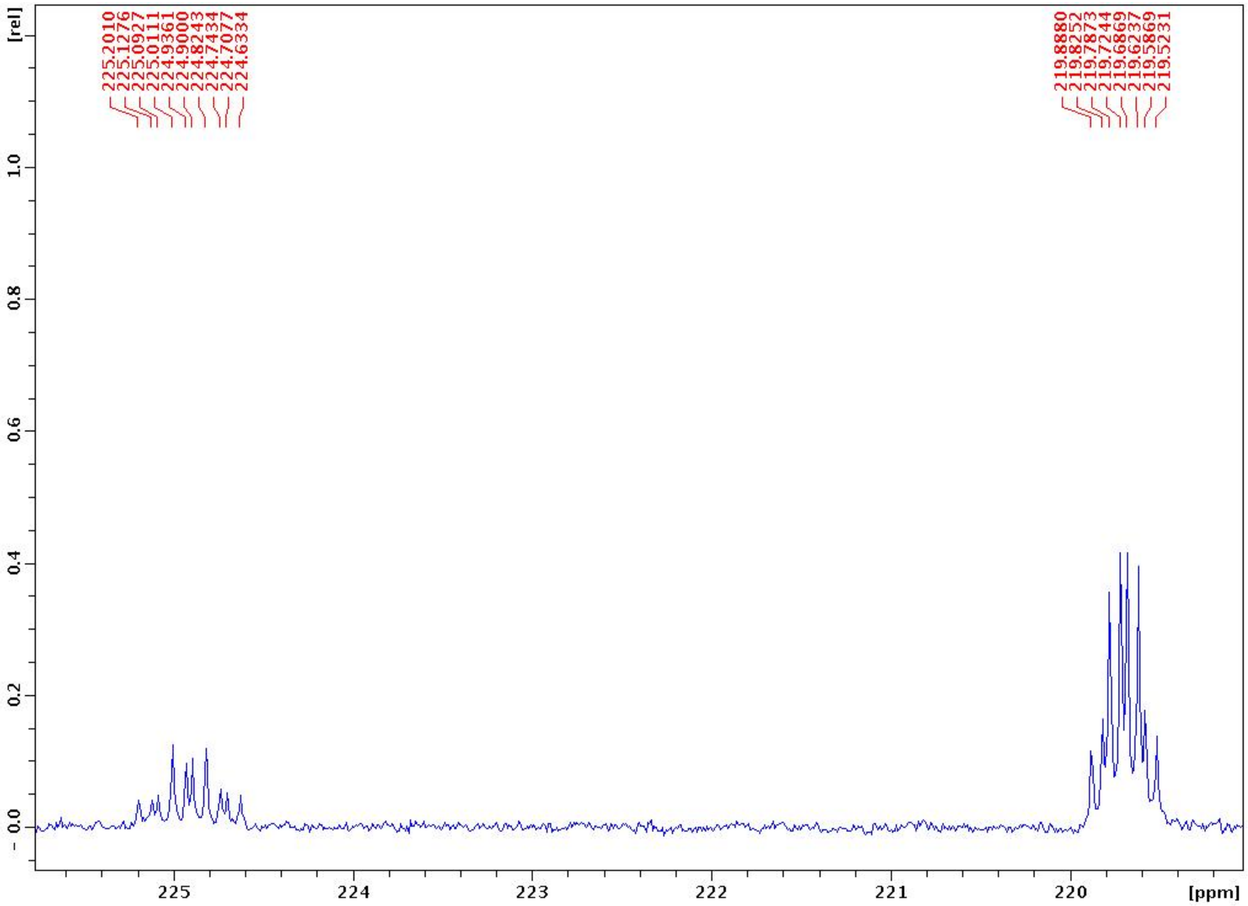

Figure S8. Partial ^{1}H-coupled ^{13}C NMR spectrum of $(^{iPr}PN^{Me}P)Fe(H)_2{}^{13}CO$ (**4a**) showing *cis*- and *trans*-isomers in C_6D_6.

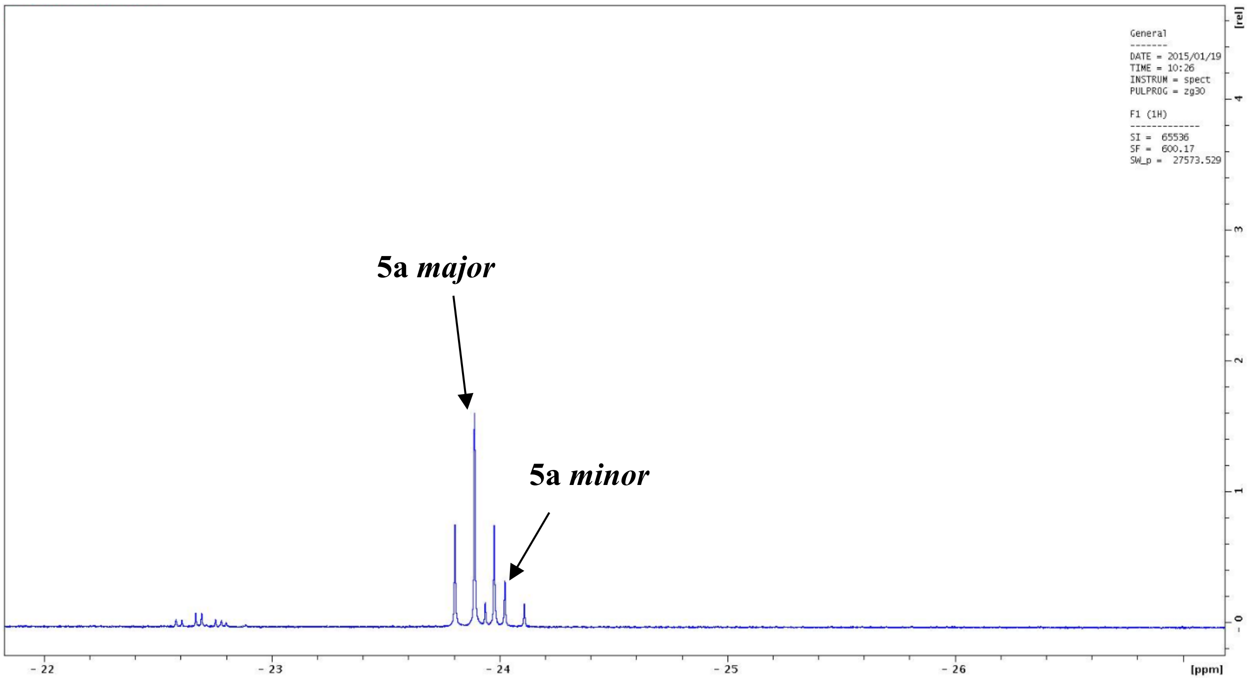

Figure S9. Partial ^{1}H NMR spectrum for **5a** showing major and minor isomers in C_6D_6. Note: **4a** present in small amount at -22.7 ppm.

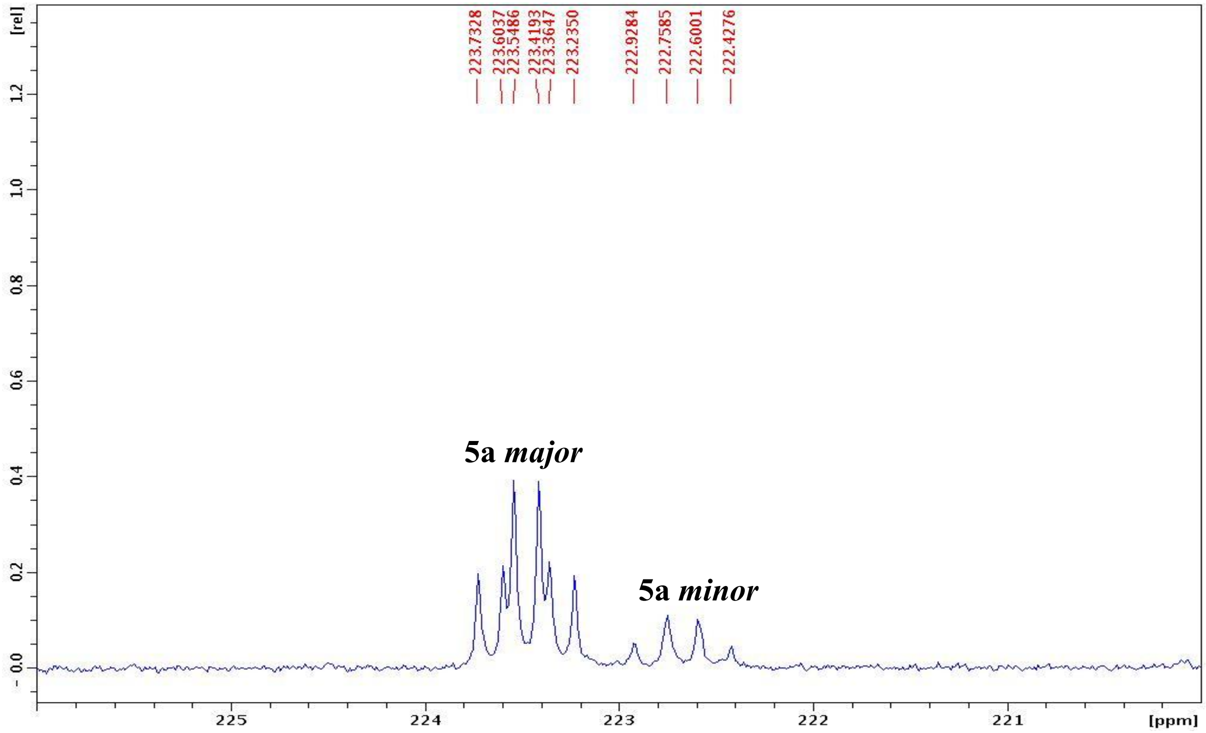

Figure S10. Partial ^{1}H-coupled ^{13}C NMR spectrum of ($^{iPr}PN^{Me}P$)Fe(H)^{13}CO(HCO_2) (**5a**) showing major and minor isomers in C_6D_6.

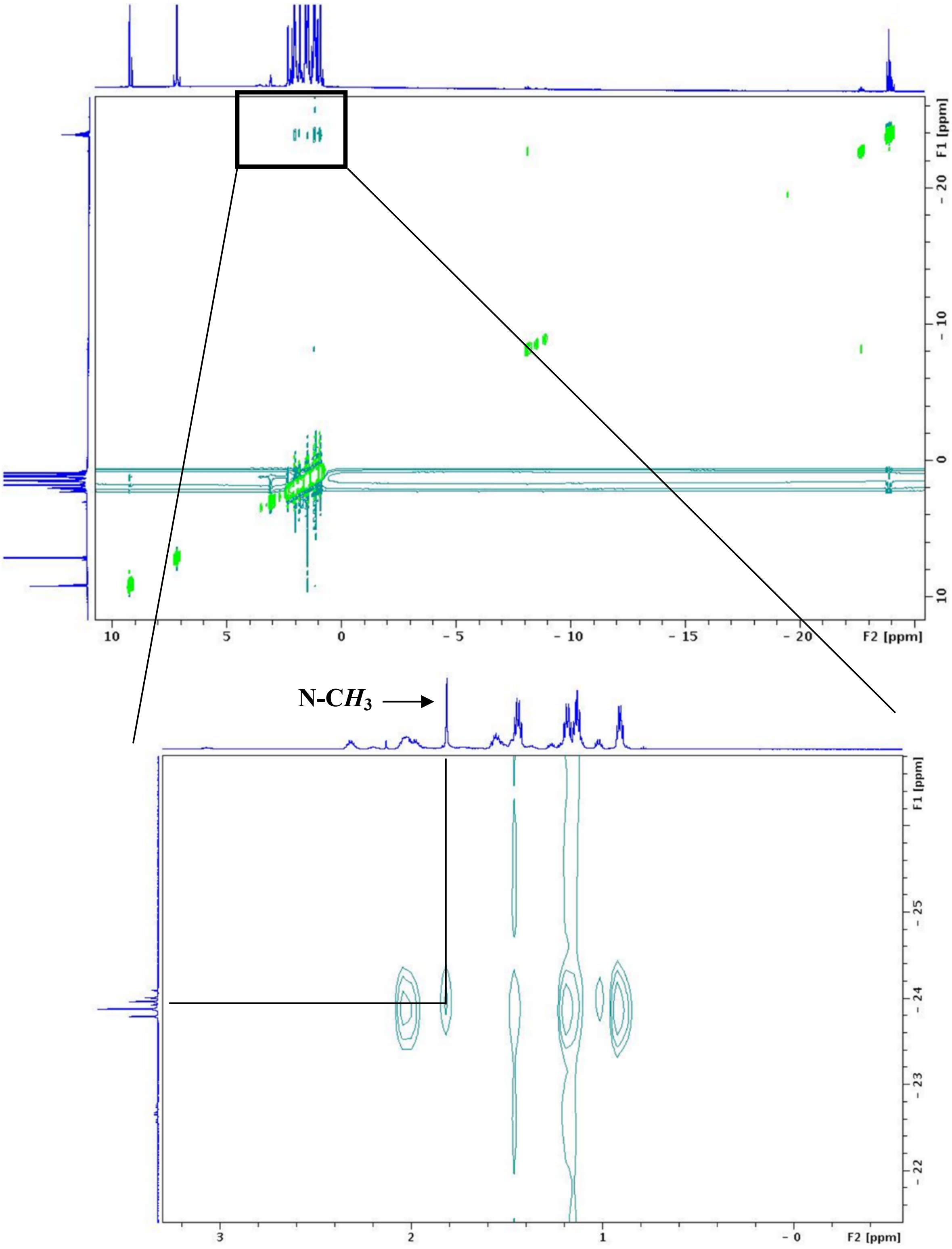

Figure S11. NOESY NMR spectrum for **5a** in C_6D_6 (mixing time 300ms at 22 °C).

Treatment of 5a with DBU, H_2 and $LiBF_4$. A J. Young NMR tube was charged with 3 mg of **5a** in approximately 1 mL of THF-*d*$_8$. 5 eq of DBU were added to the sample, then the tube was frozen at -198 °C, degassed, and 1 atm of dihydrogen was admitted. The sample was warmed to ambient temperature and monitored by ^{31}P and ^{1}H NMR spectroscopy. After 16 h, 3 eq of $LiBF_4$ was added to the sample and the dihydrogen atmosphere recharged.

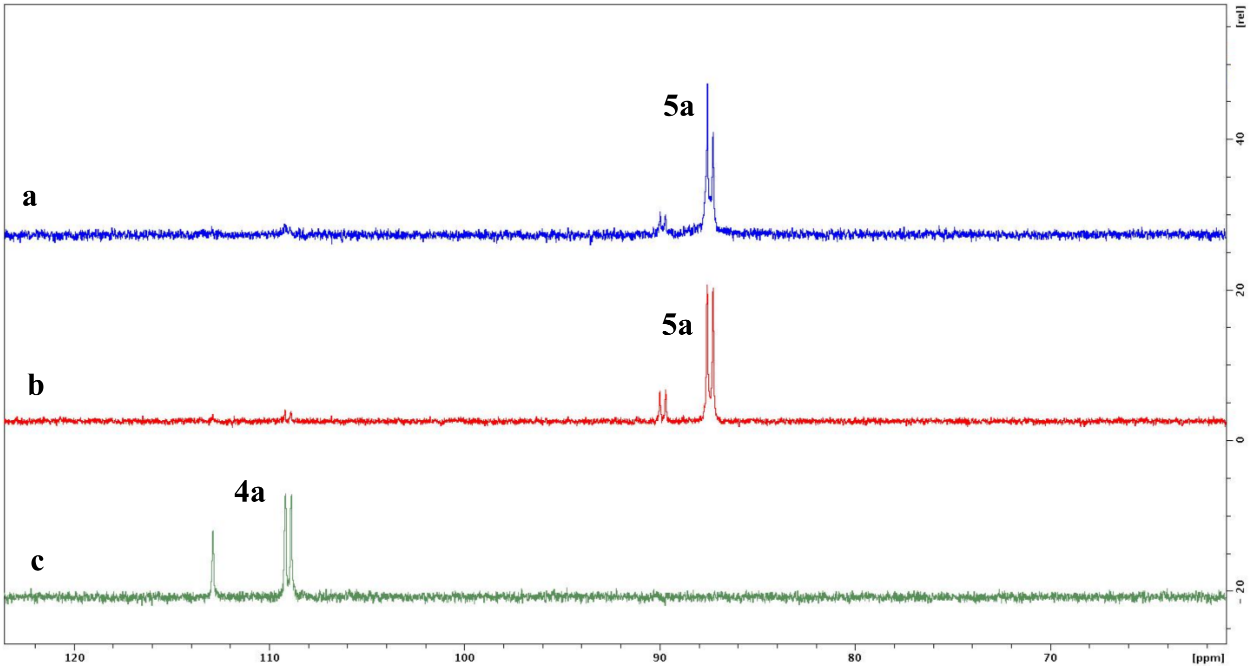

Figure S12. (a) ^{31}P NMR spectra of **5a**, (b) 16 h after addition of 5 eq DBU and 1 atm H_2, (c) 10 min after addition of 3 eq $LiBF_4$.

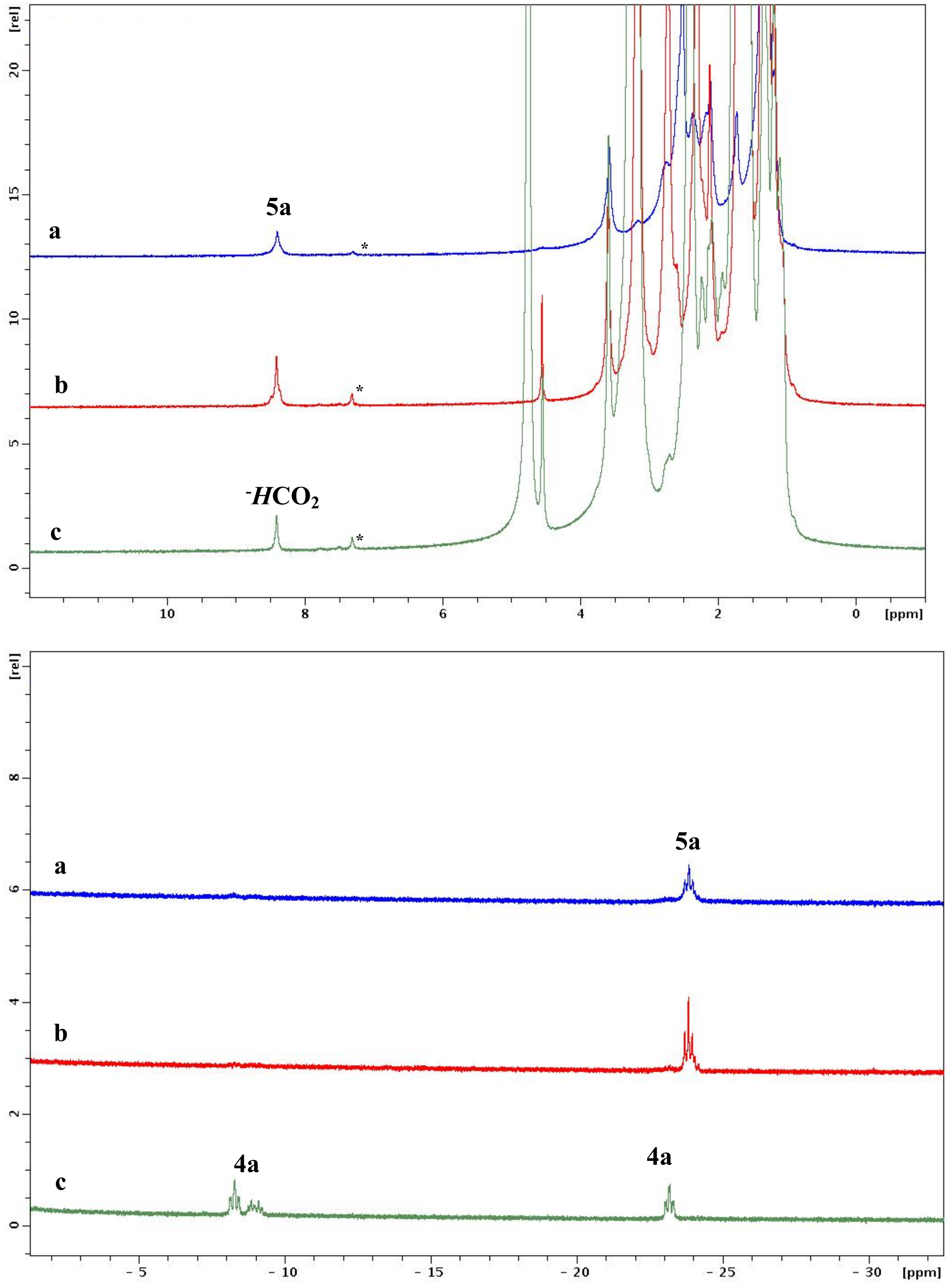

Figure S13. (a) ^{31}P NMR spectra of **5a**, (b) 16 h after addition of 5 eq DBU and 1 atm H_2, (c) 10 min after addition of 3 eq $LiBF_4$. Free formate ($^{+}HDBU$ $^{-}HCO_2$) is seen upon CO_2/H_2 addition (c). * denotes C_6H_6 from glovebox atmosphere.

Treatment of 5a with $LiBF_4$. A J. Young NMR tube was charged with 3 mg of **5a** in approximately 1 mL of THF-d_8. Then 3 eq of $LiBF_4$ was added under an N_2 atmosphere and the sample monitored by ^{31}P and ^{1}H NMR spectroscopy.

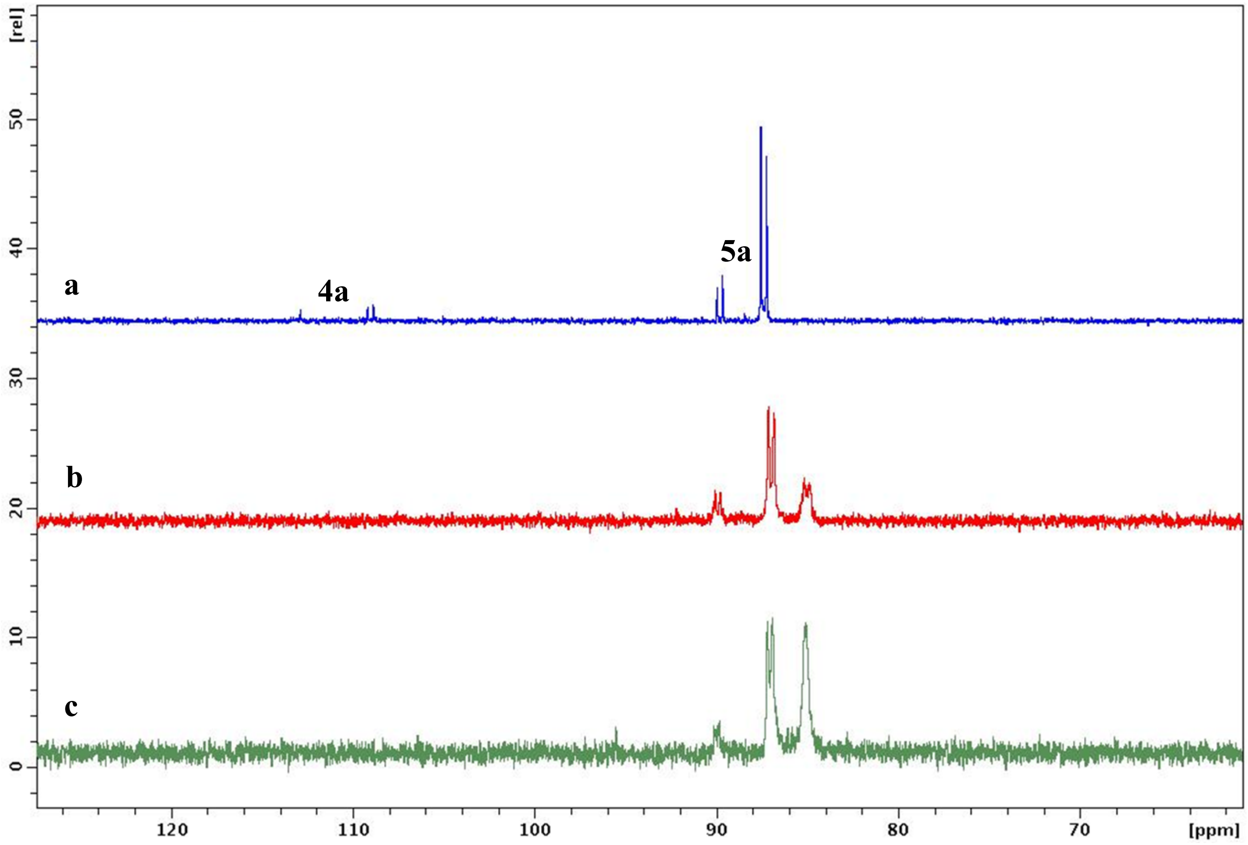

Figure S14. (a) ^{31}P NMR spectra of **5a**, (b) 10 min after addition of 3 eq $LiBF_4$, (c) 2 h after addition of 3 eq $LiBF_4$. Note: **4a** present in small amount in (a).

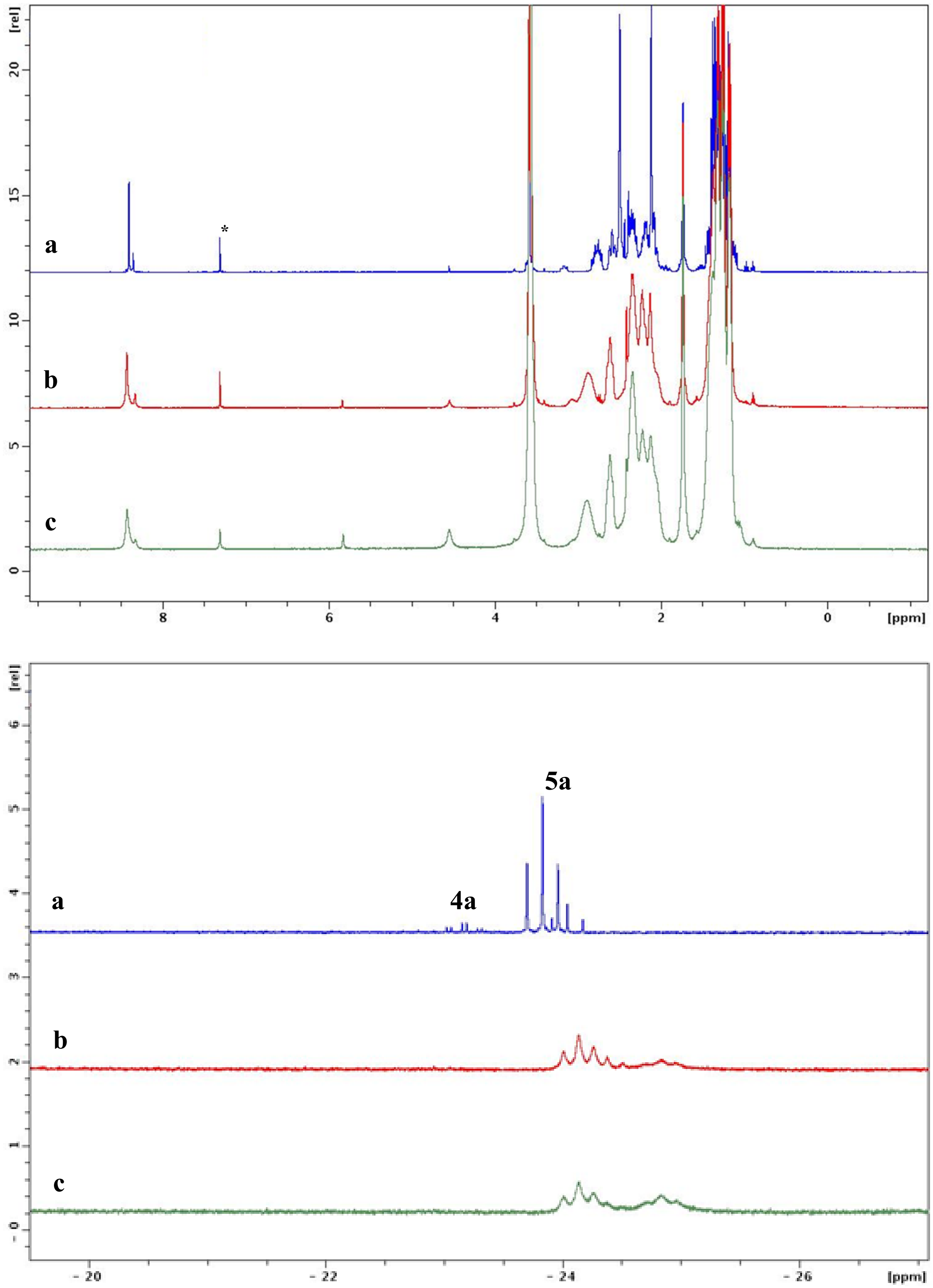

Figure S15. (a) ^{1}H NMR spectra of **5a**, (b) 10 min after addition of 3 eq $LiBF_4$, (c) 2 h after addition of 3 eq $LiBF_4$. Note: **4a** present in small amount in (a), * denotes C_6H_6 from glovebox atmosphere.

X-ray Crystallography

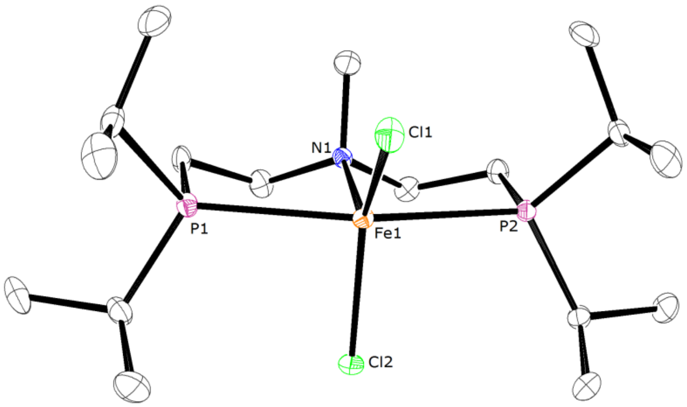

Figure S16. Molecular structures of ($^{iPr}PN^{Me}P$)$FeCl_2$ at 30% ellipsoids. Hydrogen atoms not attached to iron or boron have been removed for clarity. Selected bond lengths (Å) and angles (°): Fe(1)-P(1) 2.5749(8), Fe(1)-P(2) 2.5738(7), Fe(1)-N(1) 2.222(2), Fe(1)-Cl(1) 2.2937(7), Fe(1)-Cl(2) 2.3505(7), P(1)-Fe(1)-P(2) 162.68(3), Cl(1)-Fe(1)-Cl(2) 140.42(3).

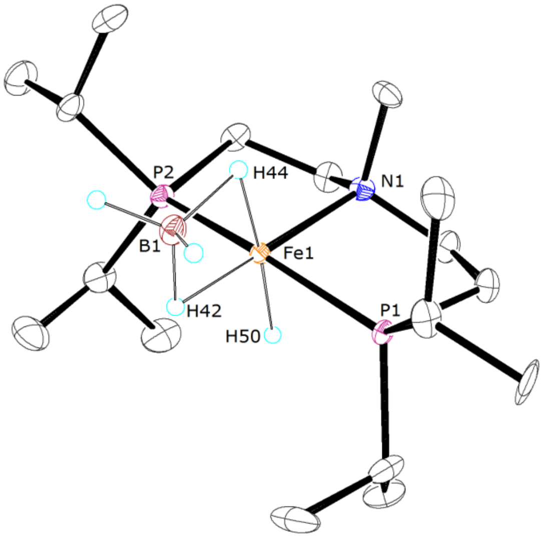

Figure S17. Molecular structures of ($^{iPr}PN^{Me}P$)Fe(H)BH_4 at 30% ellipsoids. Hydrogen atoms not attached to iron or boron have been removed for clarity. Selected bond lengths (Å) and angles (°) ($^{iPr}PN^{Me}P$)Fe(H)BH_4: Fe(1)-P(1) 2.193(2), Fe(1)-P(2) 2.183(2), Fe(1)-N(1) 2.079(2), Fe(1)-H(42) 1.62(6), Fe(1)-H(44) 1.67(5), Fe(1)-H(50) 1.58(5), P(1)-Fe(1)-P(2) 165.34(6), H(42)-B(1)-H(44) 105(4).

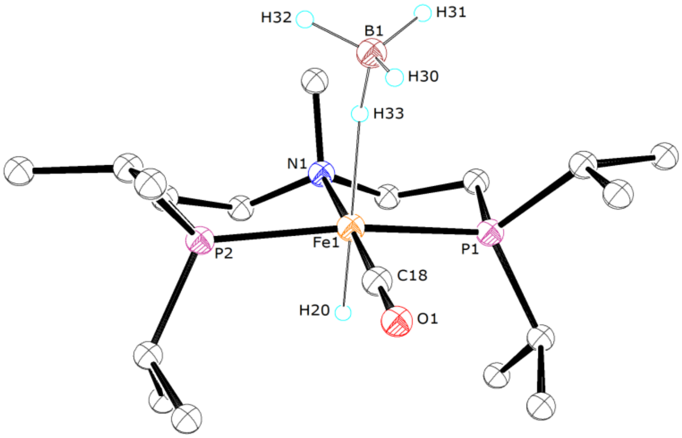

Figure S18. Molecular structure of **3a** at 30% ellipsoids. Hydrogen atoms not attached to iron or boron have been removed for clarity. Selected bond lengths (Å) and angles (°) for **3a**: Fe(1)-P(1) 2.2034(6), Fe(1)-P(2) 2.2031(7), Fe(1)-N(1) 2.136(1), Fe(1)-H(20) 1.47(2), Fe(1)-H(33) 1.81(3), Fe(1)-C(18) 1.728(2), C(18)-O(1) 1.162(2), P(1)-Fe(1)-P(2) 165.45(3), N(1)-Fe(1)-C(18) 177.55(7).

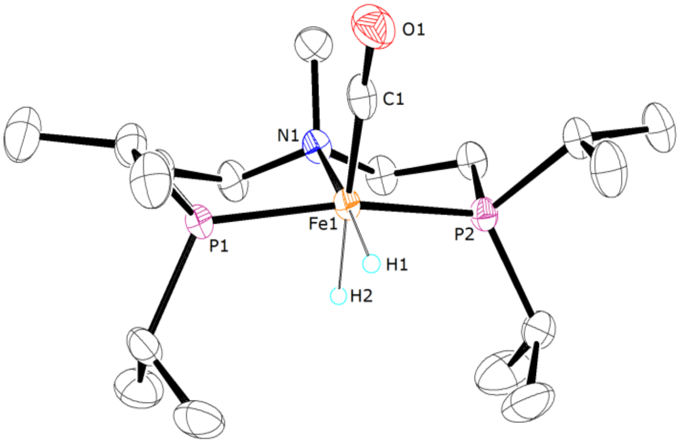

Figure S19. Molecular structure **4a** at 30% ellipsoids. Hydrogen atoms not attached to iron have been removed for clarity. Selected bond lengths (Å) and angles (°): Fe(1)-P(1) 2.1622(8), Fe(1)-P(2) 2.1583(8), Fe(1)-N(1) 2.123(2) Fe(1)-H(1) 1.51(2), Fe(1)-H(2) 1.42(3), Fe(1)-C(1) 1.748(3), C(1)-O(1) 1.134(4), P(1)-Fe(1)-P(2) 165.13(3), N(1)-Fe(1)-H(1) 171.8(9), C(1)-Fe(1)-H(2) 170(1).

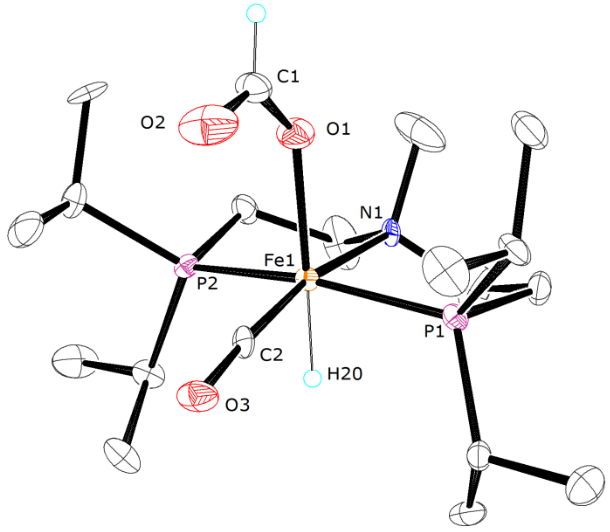

Figure S20. Molecular structure of **5a** at 30% ellipsoids. The hydrogen atom attached to iron was freely refined while that attached to C(1) was calculated. All other hydrogen atoms have been removed for clarity. Selected bond lengths (Å) and angles (°) for 3a: Fe(1)-P(1) 2.212(3), Fe(1)-P(2) 2.227(3), Fe(1)-N(1) 2.14(1), Fe(1)-H(20) 1.40(8), Fe(1)-C(2) 1.68(1), Fe(1)-O(1) 2.019(8), C(2)-O(3) 1.19(2), C(1)-O(1) 1.27(2), C(1)-O(2) 1.20(2), P(1)-Fe(1)-P(2) 163.8(1), N(1)-Fe(1)-C(2) 168.8(5); O(1)-Fe(1)-H(20) 177(4).

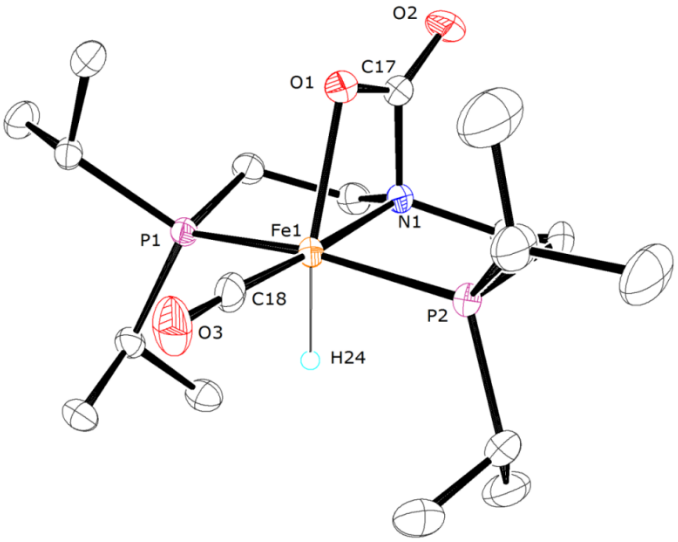

Figure S21. Molecular structure of **CO_2-1a** at 30% ellipsoids. Hydrogen atoms not attached to iron and a co-crystallized pentane molecule have been removed for clarity. Selected bond lengths (Å) and angles (°): Fe(1)-P(1) 2.2104(9), Fe(1)-P(2) 2.2051(9), Fe(1)-N(1) 2.037(2), Fe(1)-C(18) 1.720(3), Fe(1)-H(24) 1.45(3), Fe(1)-O(1) 2.113(2), C(17)-O(1) 1.262(4), C(17)-O(2) 1.212(4), C(17)-N(1) 1.513(4), C(18)-O(3) 1.167(4), P(1)-Fe(1)-P(2) 164.85(4), N(1)-Fe(1)-C(18) 175.1(1), N(1)-Fe(1)-O(1) 65.75(9), O(1)-C(17)-O(2) 131.9(3).

References

1. (a) Werkmeister, S.; Junge, K.; Wendt, B.; Alberico, E.; Jiao, H.; Baumann, W.; Junge, H.; Gallou, F.; Beller, M. *Angew. Chem. Int. Ed.* **2014**, *53*, 8722; (b) Bornschein, C.; Werkmeister, S.; Wendt, B.; Jiao, H.; Alberico, E.; Baumann, W.; Junge, H.; Junge, K.; Beller, M. *Nat. Commun.* **2014**, *5*, 4111; (c) Koehne, I.; Schmeier, T. J.; Bielinski, E. A.; Pan, C. J.; Lagaditis, P. O.; Bernskoetter, W. H.; Takase, M. K.; Würtele, C.; Hazari, N.; Schneider, S. *Inorg. Chem.* **2014**, *53*, 2133.

2. Pangborn, A. B.; Giardello, M. A.; Grubbs, R. H.; Rosen, R. K.; Timmers, F. J. *Organometallics* **1996**, *15*, 1518.

3. Sandström, J. *Dynamic NMR Spectroscopy*; Academic Press: New York, 1982.
